# Bacterial outer membrane vesicles as a candidate tumor vaccine platform

**DOI:** 10.3389/fimmu.2022.987419

**Published:** 2022-09-09

**Authors:** Shuming Wang, Jiayi Guo, Yang Bai, Cai Sun, Yanhao Wu, Zhe Liu, Xiaofei Liu, Yanfeng Wang, Zhigang Wang, Yongmin Zhang, Huifang Hao

**Affiliations:** ^1^ State Key Laboratory of Reproductive Regulation & Breeding of Grassland Livestock, School of Life Science, Inner Mongolia University, Hohhot, China; ^2^ Inner Mongolia University Research Center for Glycochemistry of Characteristic Medicinal Resources, Department of Chemistry and Chemical Engineering, Inner Mongolia University, Hohhot, China

**Keywords:** gram negative bacteria, bacteria outer membrane vesicles, cancer, tumor vaccines, immunization

## Abstract

Cancer represents a serious concern for human life and health. Due to drug resistance and the easy metastasis of tumors, there is urgent need to develop new cancer treatment methods beyond the traditional radiotherapy, chemotherapy, and surgery. Bacterial outer membrane vesicles (OMVs) are a type of double-membrane vesicle secreted by Gram-negative bacteria in the process of growth and life, and play extremely important roles in the survival and invasion of those bacteria. In particular, OMVs contain a large number of immunogenic components associated with their parent bacterium, which can be used as vaccines, adjuvants, and vectors to treat diseases, especially in presenting tumor antigens or targeted therapy with small-molecule drugs. Some OMV-based vaccines are already on the market and have demonstrated good therapeutic effect on the corresponding diseases. OMV-based vaccines for cancer are also being studied, and some are already in clinical trials. This paper reviews bacterial outer membrane vesicles, their interaction with host cells, and their applications in tumor vaccines.

## Introduction

Cancer is the main cause of death in developed countries and the second leading cause of death in developing countries (1). Currently, the main cancer treatment methods include surgical treatment, radiotherapy, chemotherapy, and small-molecule targeted therapy; however, drug resistance and the easy metastasis of tumors necessitate the development of new treatment methods (2).

In recent years, tumor vaccines have become indispensable in the treatment of many different types of cancer, including breast cancer, lung cancer, and pancreatic cancer, and have even been applied in clinical studies of multiple myeloma (3). Compared to traditional therapies, tumor vaccines are safer and more reliable for the following reasons: (1) the body’s immune system targets non-self substances, and (2) the T cell-mediated responses and antigen memory have obvious advantages during cancer recurrence (4).

Cancer vaccines can be divided into two categories, of which one is prophylactic vaccines, such as GlaxoSmithKline’s Cervarix bivalent HPV vaccine that is already on the market (5). When given to healthy people, this vaccine protects against high-risk types of HPV; however, it has no effect on those who are already infected. The other category comprises tumor therapeutic vaccines, which are mainly targeted at patients who are already sick and act to reactivate the immune system and enhance the immune response (5).

Current cancer vaccines all have some obvious disadvantages, such as low immunogenicity, and some may even introduce new cancers into patients. In recent years, bacterial outer membrane vesicles have gained attention as a backbone for new cancer treatment strategies. OMVs have already been employed as a platform for the development of tumor vaccines for several reasons: (1) the immunogenicity of OMVs is strong; (2) OMVs do not proliferate *in vitro*; and (3) OMVs can carry a target antigen and present it on the membrane surface to enhance the effect of antigen presentation (6).

Nonetheless, in order to prepare safe and efficient anti-tumor vaccines, it remains necessary for researchers to deepen understanding of tumor vaccines, the structure and generation of bacterial outer membrane vesicles, the mechanism of immune internalization and related technologies. Toward this end, this paper reviews the abovementioned subjects.

## Tumor vaccines

As cancer research has developed, more and more attention has been paid to research and applications concerning cancer vaccines. Vaccine-based approaches can work in the tumor microenvironment, and rely on the body’s recognition of non-self to attack and remove foreign substances. Cancer vaccines currently on the market can be divided by type into nucleic acid vaccines, peptide vaccines, whole cell vaccines, and live vector vaccines, detailed descriptions of which follow (5).

### Nucleic acid vaccines

Tumor nucleic acid vaccines may be based on DNA or on RNA. These vaccines rely on a gene carrier to introduce an encoded exogenous antigen into body cells, and the endogenous expression system of those cells generates the corresponding antigen and then induces a specific cellular immune response to eliminate cancer cells (7). DNA vaccines are essentially bacterial plasmids encoding tumor antigens, which contain two origins of replication: a prokaryotic origin for replication in the bacterium and a eukaryotic origin for replication in the vaccinated host (8). DNA vaccines are able to migrate to the nucleus for replication, transcription, and antigen production, and then stimulate the immune system by interacting with antigens produced on the host cell surface and their presentation on MHC class I and class II complexes (8, 9). DNA vaccines usually utilize a plasmid produced in *Escherichia coli* as the expression vector and are delivered by intramuscular injection, which can not only induce humoral immunity but also promote the proliferation and differentiation of T cells, thereby leading to cellular immune response (10).

Notably, this also leads to the host cells having a long-term memory of the corresponding response and being able to stably express antibodies so that the body can produce a lasting immune response (11). The exogenous genes used in DNA vaccines mainly encode cytokines, transcription factors, antigens, costimulatory molecules, and the like. DNA vaccines have been applied in immunotherapy to treat breast cancer, prostate cancer, cervical cancer (7), and we also summarize recent some clinical trials of DNA cancer vaccines(shown as **Supplementary Table 1**).

For example, TRIMBLE et al. used modified *HSP70* and *HPV16E7* to make a fusion DNA vaccine for use in treating HPV16-positive grade II/III cervical intraepithelial neoplasia patients; the phase I results were good, with about 33% of patients achieving complete pathological remission after 15 weeks of treatment (12). Kyriakopoulos et al. used a DNA vaccine (PTVG-AR, Mvi-118) encoding the androgen receptor ligand-binding domain (AR LBD) to treat patients with metastatic castration-sensitive prostate cancer (mCSPC). Patients would be followed for 18 months and later studies confirmed that PTVG-AR is safe and immunoreactive in mCSPC patients, and treatment may delay the time of castration-resistance (13). In addition, because mammaglobin-A is overexpressed in 40% to 80% of primary breast cancers, Tiriveedhi et al. used MAM-A DNA vaccine for phase I clinical trials; fourteen breast cancer patients with stable metastatic disease were recruited to receive the MAM-A DNA vaccine, and the results showed that the MAM-A DNA vaccine was safe and effective in eliciting MAM-A specific CD8+ T cell responses, preliminary evidence also suggests that the progression-free survival was improved and no significant adverse events were observed (14).

However, it is still not clear whether DNA vaccines cause integration of plasmid DNA into the genomes of host cells, leaving some question as to their safety. In addition, since muscle cells are not specialized antigen presenting cells, they may induce a lower level of immune response.

RNA vaccines are usually produced with a relevant mRNA as the delivery vector, and vectors can also include lipid nanoparticles, peptides and polymers. These vaccines can induce T cell immune responses similar to those provoked by DNA vaccines (7). Weide et al. injected protamine-stabilized mRNAs coding for Tyrosinase, gp100, Melan-A, Mage-A1, Mage-A3, and Survivin in 21 metastatic melanoma patients, and they use the granulocyte macrophage colony-stimulating factor as an adjuvant. At the same time, keyhole limpet hemocyanin (KLH) was added to the vaccine in 10 patients, the results showed that the frequency of Foxp3 +/CD4 + regulatory T cells was significantly decreased in patients treated with KLH, and the frequency of bone marrow suppressor cells (CD11b + HLA-DR LO monocytes) was decreased in patients who did not receive KLH, and there were no adverse events beyond grade II (15). RNA vaccine has little side effects, good immunogenicity, and the preparation process is relatively simple. In addition to being highly effective, RNA vaccines can be translated in the cytoplasm without having to be transported into the nucleus of the host cell. As the mRNA vaccines cannot be integrated into the genome sequences of body cells, insertion mutation does not occur (16). Therefore, more and more RNA tumor vaccines are being developed, and we summarize some examples of RNA tumor vaccines here (shown as **Supplementary Table 2**).

However, the stability of RNA vaccines is poor because animals have many enzymes that act on RNA, leading RNA vaccines to be easily degraded; this also limits their applications (17). And RNA itself has intrinsic immunogenicity, which can activate downstream interferon related pathway to elicit innate immunity, so it will also consume RNA to reduce the expression of specific antigens (16). Consequently, many current studies focus on improving the stability of RNA vaccines. And for now, lipid-containing ionizable nanoparticles (LNP) developed for siRNA delivery are the most widely used as mRNA delivery materials *in vivo* (18). Kranz et al. also have reported on the lipoplexes that preferentially target DCs after systemic delivery, which is a step forward for RNA vaccine delivery (19, 20). But further studies still are needed to fully address the issue of RNA vaccine delivery, and in addition, differences in mRNA preparation and differences between animal models and humans need to be overcome (20).)

### Peptide vaccines

Tumor polypeptide vaccines are mainly derived from tumor specific antigens, oncogene proteins, or related viral proteins (21), and several clinical trials have shown that tumor peptide vaccines have therapeutic effects on tumor (shown as **Supplementary Table 3**). For this vaccine, natural active peptides are widely found in animals, plants, and microorganisms, and these can be isolated and purified by relevant technologies; however, nature peptides are of relatively low abundance in their source organisms. Accordingly, the drugs on the market are mostly synthetic peptides, which have better targeting, fewer side effects, and the ability to be produced in large quantities (22). Folate receptor alpha (FR) is the peptide, and it is overexpressed in some cancers, Kalli et al. recruited patients with breast cancer or ovarian cancer who completed conventional treatment and who showed no evidence of disease to conducted the phase I trials of folate receptor alpha peptide vaccines. Patients received early low-dose cyclophosphamide, followed by vaccinating 6 times monthly, and the results showed that the vaccination was well tolerated in all patients, and vaccines induced or enhanced immunity in more than 90% the patients (23). S-288310 is also a cancer peptide vaccine of two HLA-A*24:02-restricted peptides derived from two onco-antigens: DEP domain-containing 1 and m-phase phosphoprotein 1; so Obara et al. conducted a phase I/II study of cancer peptide vaccine S-288310 in patients with advanced urothelial carcinoma of the Bladder, and the results showed that S-288310 was well-tolerated and no difference tolerated in CTL induction rate between the 1 mg (100%) and 2 mg (80.0%) Groups (24).

On the whole, numerous studies have shown that tumor polypeptide vaccines are safe and easy to make, but peptide vaccines are easily affected by the immune escape of cancer cells due to their low immunogenicity, a single epitope and easy degradation (7). To address these issues, tumor polypeptide vaccines are usually assisted by adjuvants or combined with other vaccines to enhance their immunogenicity. In addition, although the peptide anticancer vaccine has proven the ability to induce cancer-specific immune responses in multiple studies, most of the clinical responses are still limited to a single patient, so further studies are needed on the clinical adaptation of vaccines (25).

### Whole cell vaccines

Tumor whole cell vaccines generally fall into two categories: those based on tumor cells and those based on immune cells (5).Whole-cell vaccines have some advantages in cancer treatment, and some recent clinical trials have also used whole-cell cancer vaccines (shown as **Supplementary Table 4**). Vaccines based on tumor cells use the patient’s tumor cells or allogeneic cancer cells as a source of immunity, are prepared through grinding and inactivation processes, and are often used with adjuvants to enhance immunogenicity. As these vaccines are derived from tumor cells, the antigen expression is very comprehensive, and they have seen use in the treatment of many kinds of cancers (7). Eaton et al. conducted the phase I/II study on sixty patients with hormone-refractory prostate cancer using allogeneic whole-cell vaccine. The results showed that: The patient had the increases in specific antibodies, T cells and cytokines, and the vaccine was well tolerated with no major side effects (26). However, the applications of vaccines based on tumor cells are limited by the availability of autologous tumor cells and the risk that some tumor vaccines may introduce a new cancer into patients.

The most typical immune-cell-based whole cell vaccine is based on dendritic cells (DCs); this is primarily because dendritic cells are professional antigen presenting cells and extremely important in determining the intensity and direction of the immune response (5). Vaccines based on immune cells can be prepared through fusion of DCs and tumor cells, genetic modification of DCs, sensitization using tumor antigen peptides and proteins, or sensitization with exosomes of tumor cells (27). Fusion technologies for tumor cells and DCs include electric fusion, viral fusion, and chemical fusion; the fusion cells have strong immunogenicity, high specificity, and more comprehensive antigenic determinants (7, 28). Baek et al. treated 6 patients with renal cell carcinoma and 4 patients with breast cancer in a phase I/II trial with a combination of autologous dendritic cell vaccine and IL-2 adjuvant. The results showed that the combination of dendritic cell tumor vaccine and IL-2 was well tolerated by patients without serious side effects (29).

However, at present, the application of whole-cell vaccine still has great limitations. The therapeutic effect of whole-cell vaccine on immunocompromised patients is poor, and the immune function of many patients participating in clinical trials is defective, which also makes the evaluation and development of whole-cell tumor vaccine have certain limitations (30). So researches on whole-cell vaccines still need to be more comprehensive and in-depth.

### Live vector vaccines

Live vector vaccines can be divided into bacterial vaccines and viral vaccines, which mainly use attenuated or very weak viruses and bacterium as carriers and their DNA encodes the corresponding tumor antigens; then the vector is injected into the body to achieve antigen presentation and trigger an immune response (5). Regarding bacterial vaccines against cancer, many bacterium are in a highly metabolically active state in tumor cells and they can enter tumors in remote areas and are not sensitive to chemotherapy drugsthey, so they are considered as potential candidates for anticancer drugs/gene-based vaccines to treat tumors (31). Maciag et al. fused live attenuated listeria and Listeriolysin O (LLO) to produce a vaccine secreting the *HPV-16E7* antigen, but severe fever symptoms occurred during treatment (32). As this poses a risk for some patients with low immunity, the safety of patients cannot be fully guaranteed. Therefore, for bacterial vaccines, how to reduce the side effects caused by vaccines during the treatment process is the key to the development of bacterial vaccines.

For viral vaccines, adenovirus and poxvirus are widely used at present, both of which have good immunogenicity and reliable safety; the vaccines have been applied in the clinical treatment of prostate cancer, lung cancer and so on (5, 7) (shown as **Supplementary Table 5**). Since a virus is itself invasive, it can effectively infect DCs, improve the efficiency of antigen presentation, and resist inactivation by complement (33). And for the time being, virus vector-Car T. Targeting transgene expression to DCs has become a promising approach for guiding the immune system towards immunity or tolerance, which is able to be achieved on a transcriptional level by utilizing DC-specific promoters or by retargeting the tropism of the virus vectors (34, 35). TG4010 is a recombinant viral vaccine, which can express the tumor-associated antigens MUC1 and interleukin-2, and this vector is based on the modified and attenuated strain of vaccinia virus. Ramlau et al. conducted a phase II study of Tg4010 in association with chemotherapy in patients with stage III/IV Non-small cell lung cancer, and recruited 68 patients with stage IIIB/IV non-small cell lung cancer. The results showed that the combination of TG4010 with standard chemotherapy in advanced non-small cell lung cancer is feasible (36).

However, because the vector carries some of its own viral antigens in addition to the target antigen, the presence of existing virus-related immune memory in the body will reduce the immune response against the target antigen. To address this, researchers usually adopt the method of multiple viral vaccines and multiple immunization, thereby strengthening the relevant immune response.

All told, although studies on anti-tumor vaccines are getting more and more in-depth, and the diversity of tumor vaccines is increasing, all types of vaccines have some disadvantages, and many fall out of the running at the clinical trial stage. Therefore, it is necessary to explore a new tumor vaccine platform, and bacterial membrane vesicles provide a new direction for such exploration.

## Bacterial outer membrane vesicles

### Structure and components of bacterial outer membrane vesicles

**Figure 1:**
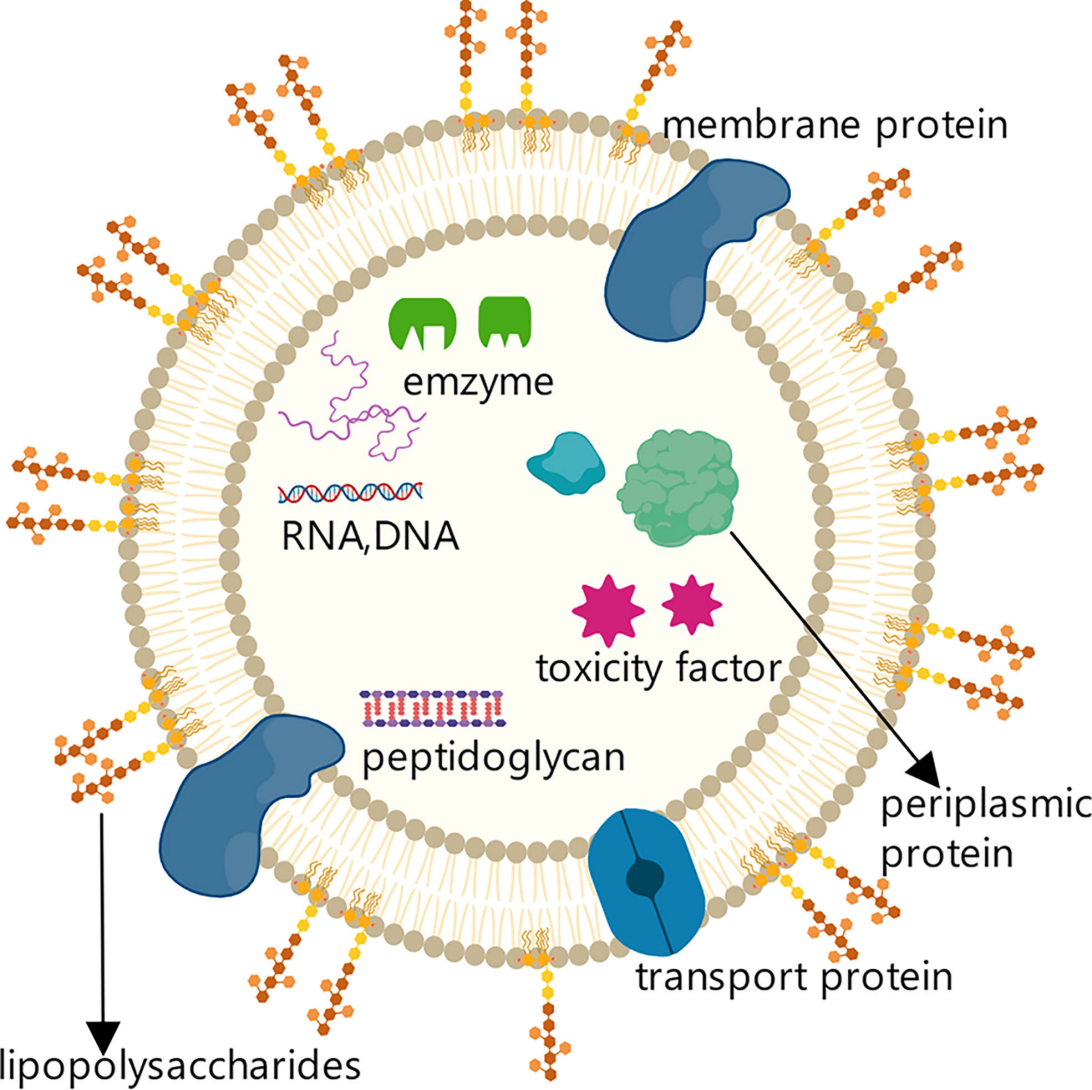
Structure and components of OMVs secreted by Gram-negative bacteria. Structure and components of OMVs secreted by Gram-negative bacteria. OMVs secreted by Gram-negative bacteria are lipid-based spherical vesicles that feature lipopolysaccharides (LPS), membrane proteins, transporters, and other substances on the membrane surface.

The first observation of the outer membrane vesicles (OMVs) produced by Gram-negative bacteria can be traced back to the 1960s; such membrane vesicles were subsequently also identified in Gram-positive bacteria and archaea (37). OMVs are double-layer membrane vesicles that typically have a lipid-based spherical structure with a diameter of 20-250 nm. They contain bacterial outer membrane and periplasmic space components, such as several types of outer membrane proteins, lipopolysaccharide (LPS), a variety of cytotoxic factors related to cell adhesion and invasion, periplasmic space proteins, DNA, RNA, and enzymes (**Figure 1**). These components enable OMVs to affect a variety of bacterial biological processes, including nutrient intake, information transfer, DNA transfer, and the transport of cell metabolites and other substances (37, 38).

Studies have shown that the ability of OMVs to uptake nutrients can be reflected in their absorption of iron and zinc from plasma, and the OMVs secreted by *Neisseria meningitidis* have been found to contain large quantities of FetA, ZnuA, and proteins that transport iron and zinc ions (39). In addition, some macromolecules can serve as common resources among various bacterium after the digestion and degradation of OMVs, which can provide more favorable conditions for bacterial survival (40).

OMVs can also act as a bridge for information exchange and assist the infection of host cells. Namely, OMVs can be recognized by the host immune system through surface and lumenal proteins such as toxoid factors, DNA, RNA and other components (41), and that recognition can inhibit the immune response, promoting infection and triggering inflammation (42).

Ultimately, the physiological characteristics and functions of OMVs can allow them to effectively deliver antigens to antigen presenting cells (APCs). Moreover, weak immunogenicity of antigens and need for adjuvants are rarely concerns for OMV-based vaccines. However, surface levels of lipopolysaccharide and other substances need to be reduced on OMVs to avoid potentially adverse inflammatory reactions (6).

### Formation of bacterial outer membrane vesicles

**Figure 2:**
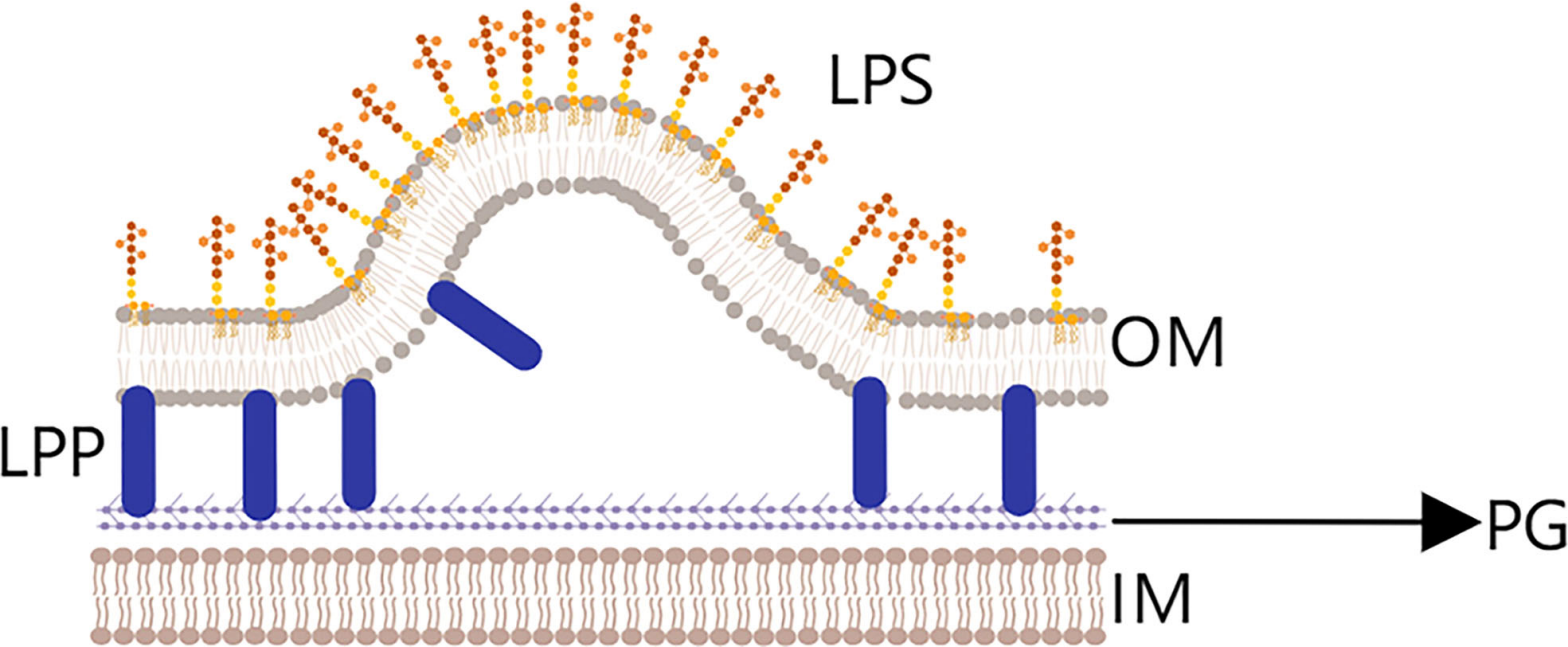
Membrane cross-linking regulation hypothesis.Lipoprotein (LPP) is present in the periplasmic space between the outer membrane layer (OM) and peptidoglycan layer (PG) layers, and plays an important role in the connection fixing the two layers. If LPP is degraded, the connection will be weakened, and outer membrane vesicles will begin to be released.

There are three main hypotheses for the generation of OMVs (**Figure 2**). (1) In the membrane cross-linking regulation hypothesis (43), lipoprotein (LPP) is present in the periplasm of Gram-negative bacteria and acts to connect the outer membrane region with the peptidoglycan layer. If LPP is deconstructed, the connection between the two layers will weaken or disappear, which could make the outer membrane bulge and lead to OMV production and release. Notably, Deatherage (44) observed a significant increase in the number of OMVs produced in the absence of outer membrane protein A (OmpA), which normally interacts with the lipoprotein complex.

**Figure 3:**
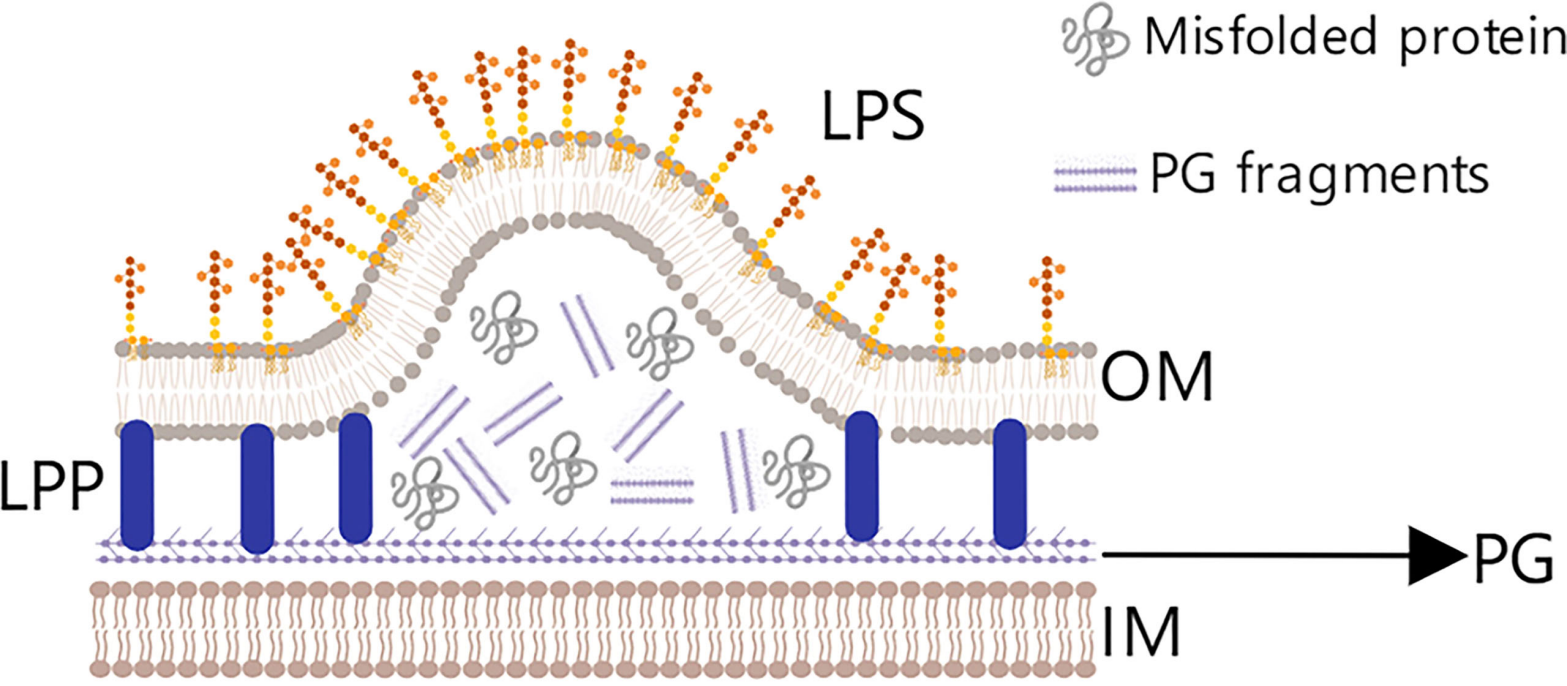
The pressure hypothesis. Peptidoglycan fragments and misfolded proteins are abundant in the periplasmic space of Gram-negative bacteria, and their aggregates put pressure on the outer membrane (OM), ultimately causing the OM and PG to separate and OMVs to be released.

**Figure 4:**
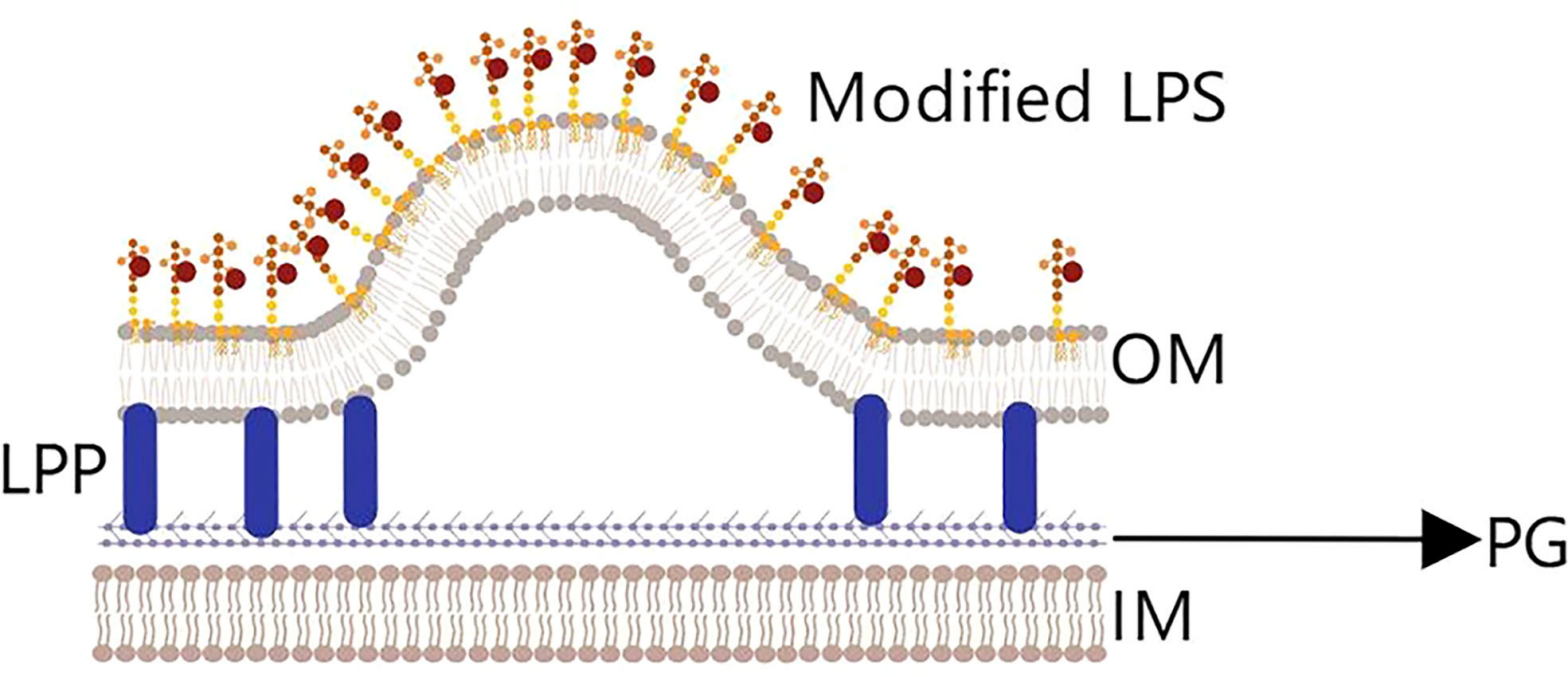
The lipid enrichment hypothesis. Adding phospholipids or modifying lipopolysaccharides (LPS) will change the curvature of the outer membrane and cause OMVs to be released. Temperature variation and the level of LPS acylation also impacts the generation of OMVs.

(2) The pressure hypothesis, also called the cell stress hypothesis, posits that the periplasmic space in bacteria contains many segments of peptidoglycan and misfolded proteins (**Figure 3**). These substances usually aggregate and put pressure on the plasma membrane, which causes the outer membrane and peptidoglycan layer to gradually separate and hence produce and release OMVs (45). Bacterium can release these useless or even harmful substances to reduce the pressure in the interest of survival. This effect can be observed by treating *Pseudomonas aeruginosa* with epoxy floxacin; in the event of DNA damage, the bacteria initiate an emergency rescue mechanism that increases the number of OMVs released so that it is more conducive to fighting the injury and harsh living environment (46).

(3) Finally, the lipid enrichment hypothesis (45) concerns the composition of the cell membrane, which mainly includes lipids and proteins (**Figure 4**). Studies have found that adding phospholipids or modified lipopolysaccharides to the bacterial outer membrane could change the curvature of the membrane and increase the number of OMVs released (47). In addition, changing the temperature to affect membrane fluidity and increasing or decreasing LPS acylation level both impact the generation of OMVs (48, 49).

## Interaction of host cells and OMVs

The OMVs released by Gram-negative bacteria not only play important roles between bacterium but also in the interaction between bacterium and host cells. The physiological characteristics of OMVs allow them to trigger an immune response even in the case where there is no contact between the originating bacteria and host cells; this property inspired the proposal to use OMVs as a vaccine platform. In addition, OMVs contain LPS and lipoproteins that can activate the programmed death signal in a host cell during their interaction, triggering cell death (50).

### Immune reaction

If we are to develop anti-tumor vaccines based on the OMV platform, it is important to reduce or even eliminate their side effects on the body while enhancing their efficacy; this requires fully understanding the interactions of OMVs and host cells. OMVs can trigger a response in the biological immune system, which is caused by recognition of the receptors on the OMV membrane surface by somatic cells; the OMVs are judged to be “non-self”, and body’s immune system consequently activated.

**Figure 5:**
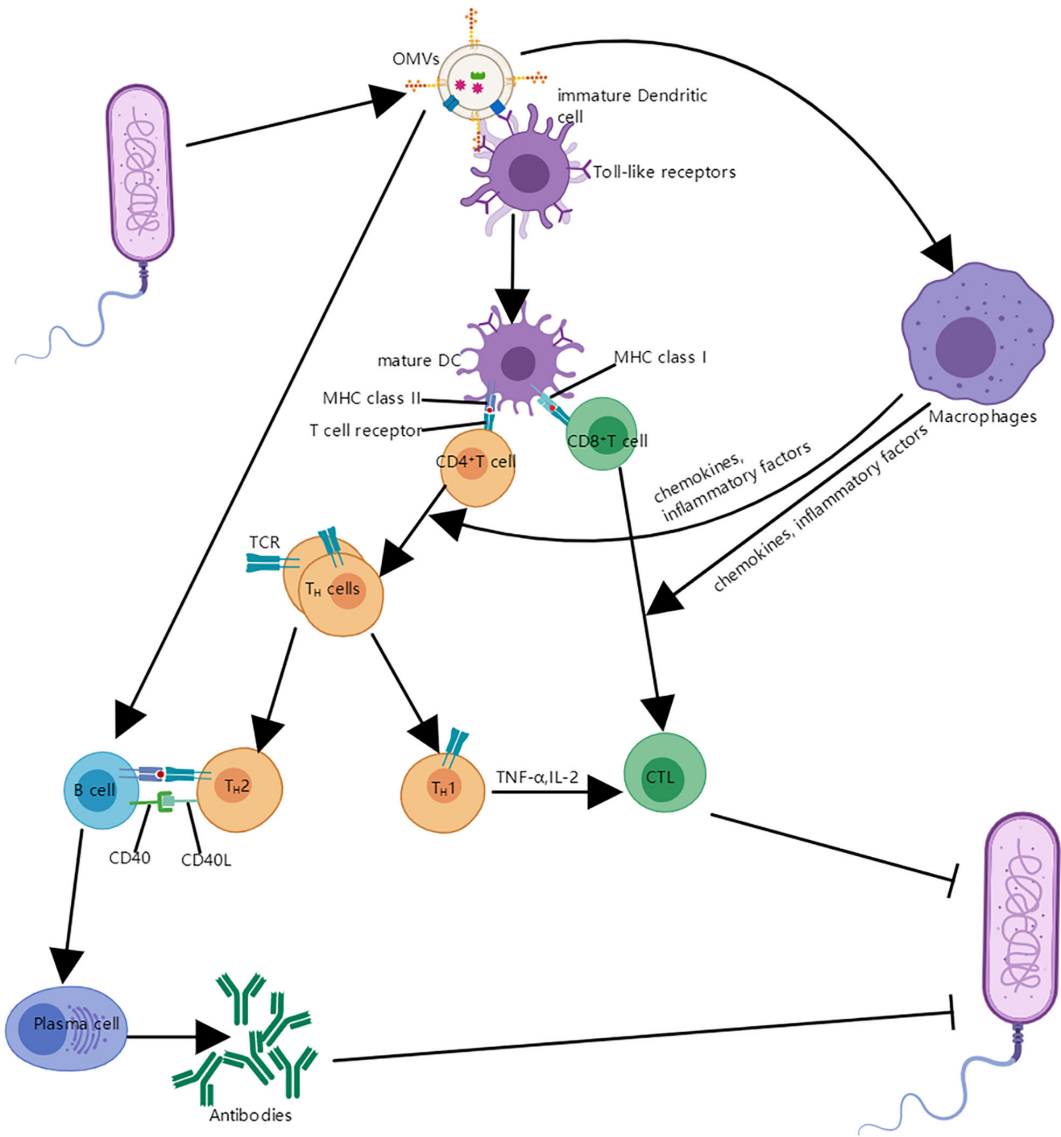
The interaction between OMVs and immune system. OMVs interact with immature DCS and are internalized. Mature DCs can process antigens and then present antigen epitopes on their surface in the form of the MHC complex; the modified antigen is recognized by the T cell antigen receptor on the CD4 T cell and CD8 T cell surface, which leads to the helper T cells and cytotoxic T lymphocyte (CTL). In addition, OMVs can also interact with B cells and proliferate into plasma cells and produce antibodies with the assistance of T_H_2 cells; OMVs can also be absorbed by macrophages, which makes expression of cytokines and chemokines increase and it is helpful for the proliferation and differentiation of T cells.

This process generally requires uptake of OMVs by specialized antigen presenting cells, such as DCs, which can process antigens and then present antigen epitopes on their surface in the form of the MHC complex; subsequently, the modified antigen is recognized by the T cell antigen receptor on the T cell surface, and T cells are activated to trigger an immune response involving cytotoxic T cells and helper T cells (5); OMVs can also interact with macrophages and cause macrophages to produce cytokines and chemokines, which can act on CD4 T cells and CD8 T cells (51, 52). In addition, helper T cells contain T_H_1 cells and T_H_2 cells; CD40 ligand (CD40L) on these activated T_H_2 cells interacts with its cognate receptor on B cells, which can promote the proliferation and differentiation of B cells into plasma cells and produce corresponding antibodies (52, 53) (**Figure 5**).

In addition, OMVs can interact directly with immune cells and epithelial cells through pattern recognition receptors, which can induce production of chemokines and cytokines to activate an immune reaction (54). For example, OMVs produced by *Vibrio cholerae* or *Actinobacillus* can be recognized by human epithelial cell receptors and activate NOD1 and NOD2-dependent inflammation. At the same time, host epithelial cells that have internalized OMVs are degraded through NOD1-dependent autophagy to stimulate the inflammatory response (55).

Besides the above, OMVs released by some bacteria such as *Pseudomonas* and *Tuberculosis bacilli* can also interact with TLR4 to activate inflammatory reactions (56). Similarly, OMVs from *Porphyromonas gingivalis* are able to activate TLR2, TLR4, TLR7, TLR8, and TLR9 (57).

Finally, numerous studies have shown that OMVs can cross the gut mucosal barrier through a sulfatase–dependent mechanism, and that subsequent contact with intestinal epithelial cells and intestinal macrophages results in presentation of OMV antigens, eventually leading to intestinal inflammation (58).

### Internalization

**Figure 6:**
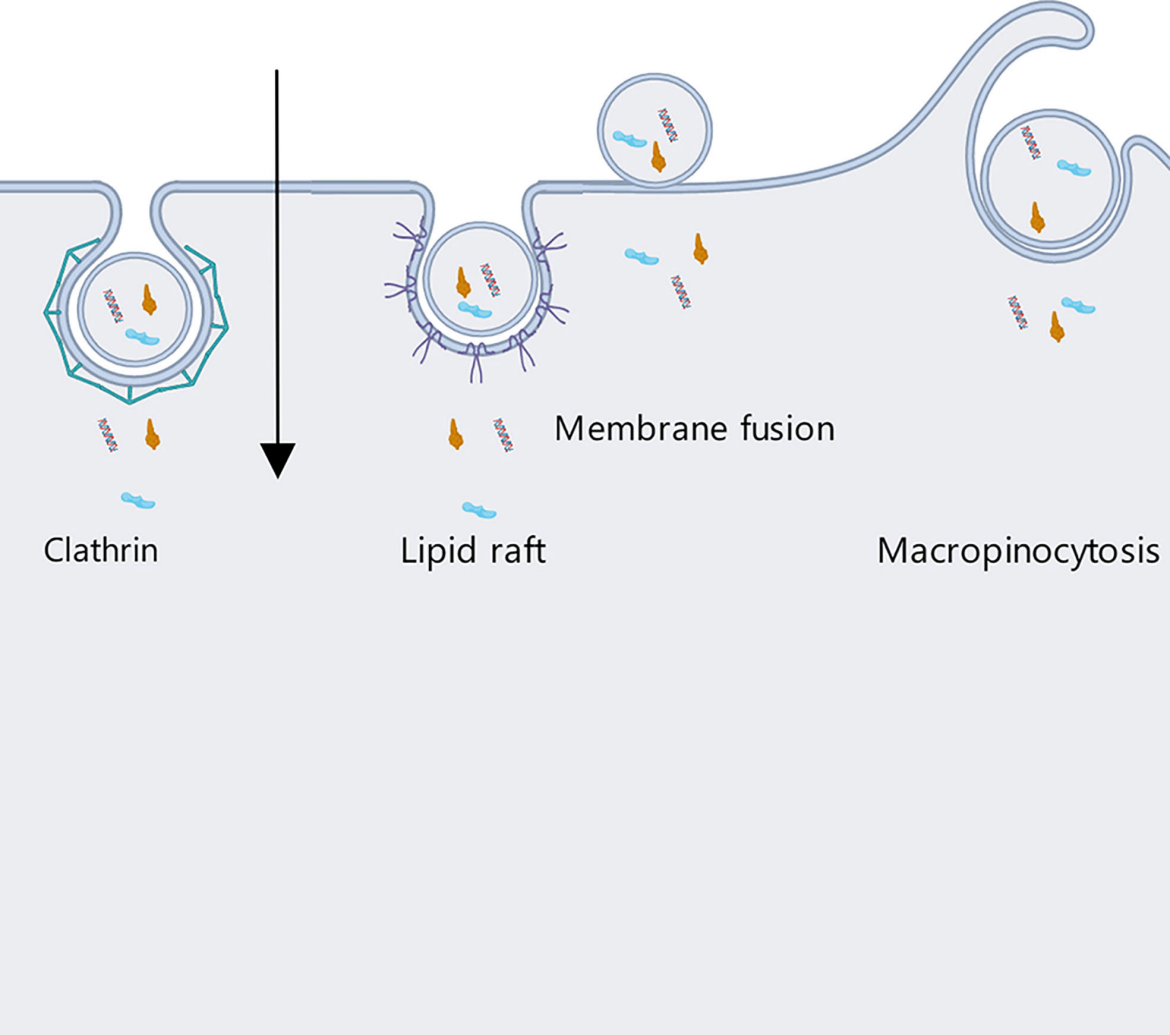
Host cells internalize OMVs secreted by Gram-negative bacteria through four different pathways: clathrin, caveolin and lipid raft-mediated endocytosis, micropinocytosis, and membrane fusion.

For nonphagocytic cells, internalization of OMVs can be achieved by means of four different pathways: clathrin, caveolin and lipid raft-mediated endocytosis, macropinocytosis, and membrane fusion pathways (**Figure 6**) (59).

(1) In clathrin-mediated endocytosis, first dynamin is utilized for budding off, then clathrin pits are formed and the OMVs are internalized. The materials contained in the vesicles can then either be returned to the cell surface or delivered directly to lysosomes for degradation (60).
The process of clathrin-mediated endocytosis is initiated by the recognition of bacterial virulence factors carried on OMVs by receptors in the host cell membrane (59). Notably, clathrin-dependent endocytosis can be applied to particles of up to around 120 nm in diameter, but OMV diameters range between 20 nm and 250 nm (54).
(2) Caveolin and lipid raft-mediated endocytosis functions through the invagination of a membranous region rich in cholesterol, pit protein, and sphingolipids; such regions are about 80 nm in diameter (61). Sharpe et al. found that OMVs and caveolins could colocalize, and that OMV internalization was mediated by caveolins (62). This process is about five times slower than clathrin-mediated internalization, but is effective at dispatching OMVs into the cell cytosol (60).
Similarly, aggregation of lipid raft regions rich in cholesterol and sphingolipids can cause invagination of the plasma membrane (here the operative diameter is about 90 nm) (54), which phenomenon can also mediate the internalization of OMVs by host cells.
(3) Unlike the methods above, macropinocytosis is effective for OMVs above 200 nm in diameter. Experiments conducted by Weiner et al. showed macropinocytosis to produce pleats of over 200 nm in diameter, allowing the cells to internalize OMVs (63). Similarly, Amano et al. demonstrated that macropinocytosis can be used to internalize vesicles of 1 micron in diameter (64).
(4) Finally, the membrane fusion pathway has been suggested as a mechanism for OMV uptake by host cells, despite the fact that OMV and host cell plasma membranes differ in structure. Bomberger et al. labeled OMVs from Pseudomonas aeruginosa with the self-quenching fluorescent dye Rhodamine R18 and found that after adding the OMVs to the culture media of host epithelial cells, the dye was dequenched and fluorescence significantly increased, indicating fusion of the OMV and host membranes (65).

Ultimately, OMVs have diverse ways of entering host cells, with uptake mechanisms varying among the different kinds of OMVs and even among vesicles produced by the same bacterium. It is necessary to develop a method that can quantifiably and dynamically assay uptake pathways and the links between OMVs and host cells (60) so that we can develop a better understanding of the relevant mechanisms, which will be important in the development of OMV-based anti-tumor vaccines and improvement of their treatment efficiency.

## Bacterial outer membrane vesicle-based tumor vaccines

Anti-tumor vaccines based on OMVs are mainly developed by using genetic engineering technology to cause a foreign protein to be expressed in the vesicle lumen or on its membrane surface; the antigen can then trigger a desired immune response without affecting the original immunogenicity and with avoidance of side effects. At present, only meningococcal related OMVs vaccine is intensively studied, and OMVs tumor vaccine still needs more comprehensive and in-depth research (shown as **Supplementary Table 6**). But a relatively mature method is to fuse an antigen with a protein from the OMV, such as CytolysinA (ClyA) and hemoglobin protein (Hbp); the resulting chimeric protein can be expressed on the OMV membrane. Researchers can also inject antigens, small RNAs, and other substances into the OMV cavity to achieve immune effects or to silence relevant genes and kill cancer cells.

### Tumor vaccines based on ClyA fusion proteins

**Figure 7:**
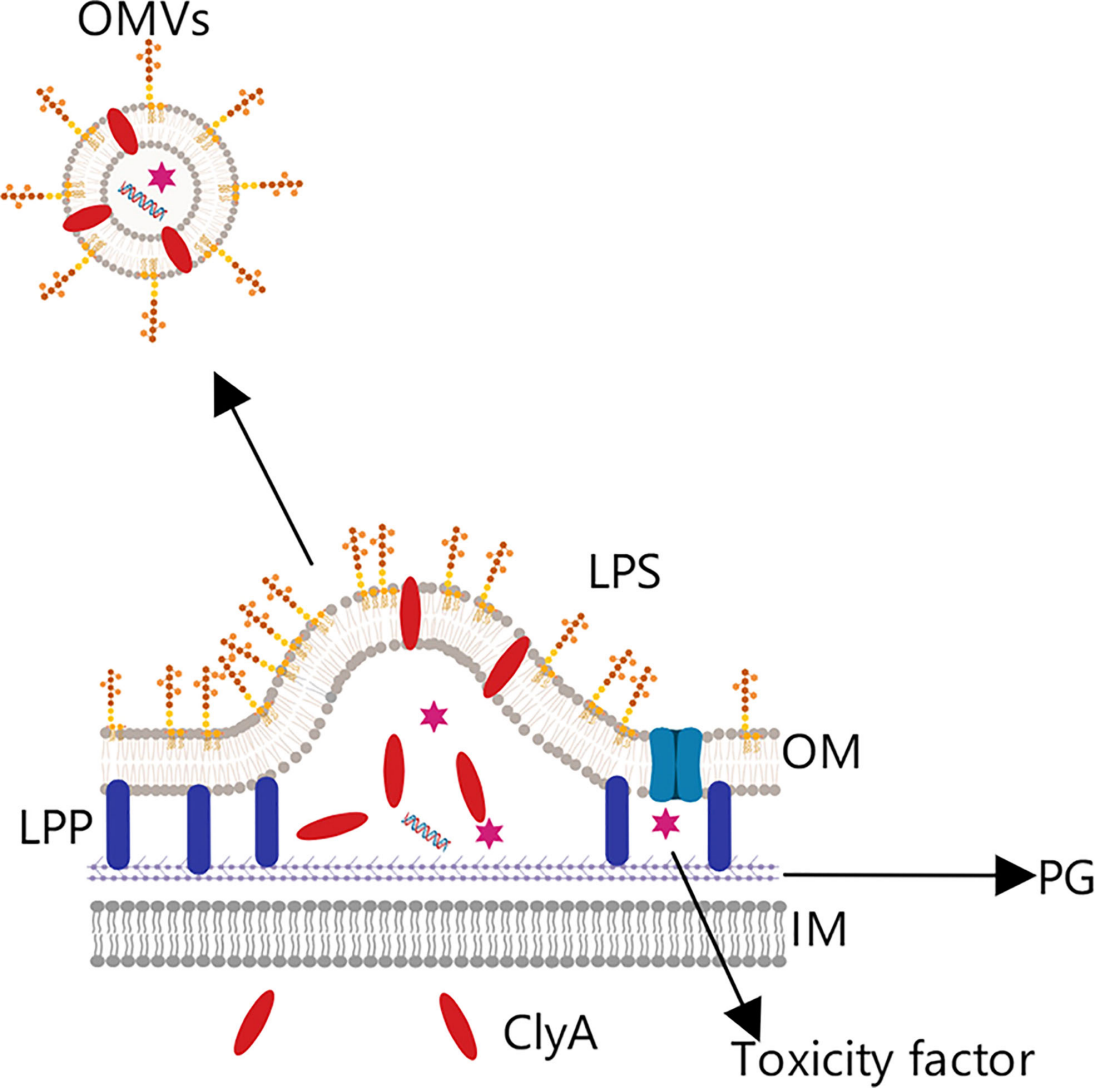
OMV tumor vaccine based on the Cytolysin A protein. Cytolysin A (ClyA) can enter the periplasmic space from the intracellular, where it then binds to the outer membrane phospholipid bilayer due to its hydrophobic structure. This characteristic was exploited by combining the C-terminus of ClyA with a specific antigen to construct a chimeric protein expressed on the OMV surface, from which was prepared the corresponding tumor vaccine.

ClyA is abundant on the OMV membrane surface and contributes to the infection process (**Figure 7**) (66). David et al. took advantage of this, fusing green fluorescent protein (GFP) and ClyA to produce a chimeric protein, ClyA-GFP, that could be localized to the OMV membrane (67). Experimental results in mice yielded a significantly higher antibody titer in the group treated with chimeric protein than in other groups, and the immune response intensity obtained with the chimeric protein was equivalent to that from the purified antigen protein with adjuvant (68). This indicates that recombinant OMVs can effectively trigger the immune response of mice when acting as an exogenous protein carrier, and furthermore that engineered OMVs can be used to strengthen the immunogenicity of weakly antigenic proteins, functioning similarly to a vaccine adjuvant.

In addition, David et al. indicated that one of the great advantages of producing recombinant OMVs is that the vesicles can be purified by simple supercentrifugation, avoiding the extremely complex purification process required for some vaccines (68).

In another study, ClyA was fused with the EGF receptor 2 antibody to form a chimeric protein presented on the membrane surface of OMVs, in addition, a small RNA molecule was imported into the OMVs by electroporation. The recombinant OMVs were demonstrated to successfully present the small RNA, which effectively silenced the related gene, promoted apoptosis, and induced significant cytotoxic effects (5, 69).

Besides the above, some researchers have taken advantage of the characteristics of the ClyA protein to simplify the antigen display process, developing the Plug-and-Display system. The main principle of the system is that ClyA binds to the catcher protein SpC/SnC, and the specific antigen protein binds to the tag protein SpT/SnT; subsequently, the catcher and tag proteins form a peptide bond, which can simplify the antigen display process and allow presenting of different tumor antigens on the OMV surface (70). In this study, ClyA-ovalbumin fusion protein (CO) was prepared by gene recombination technology. The results showed that CO OMVs could activate mouse bone marrow-derived dendritic cells and induce immune response. Then, the epitope TRP2180-188 (SVYDFFVWL) of tyrosinase-associated protein 2 (TRP2) in mouse B16-F10 melanoma model was used for analysis. The ClyA-SpC-SpT-TRP2 OMVs prepared by Plug-and-display system, ClyA-none OMVs (CN OMVs), SnT-TRP2 alone and SnT-TRP2+CN OMVs were used for immune comparison test. It was found that the plug-and-display treatment group had the most effective anti-cancer effect, and the subsequent immunological comparative experiments also showed that: the plug-and-display system can simultaneously present OVA257-264 and OVA++223-339 or OVA257-264 and TRP2 and stimulate good immunotherapeutic effect (70).

### Tumor vaccines based on Hbp fusion proteins

**Figure 8:**
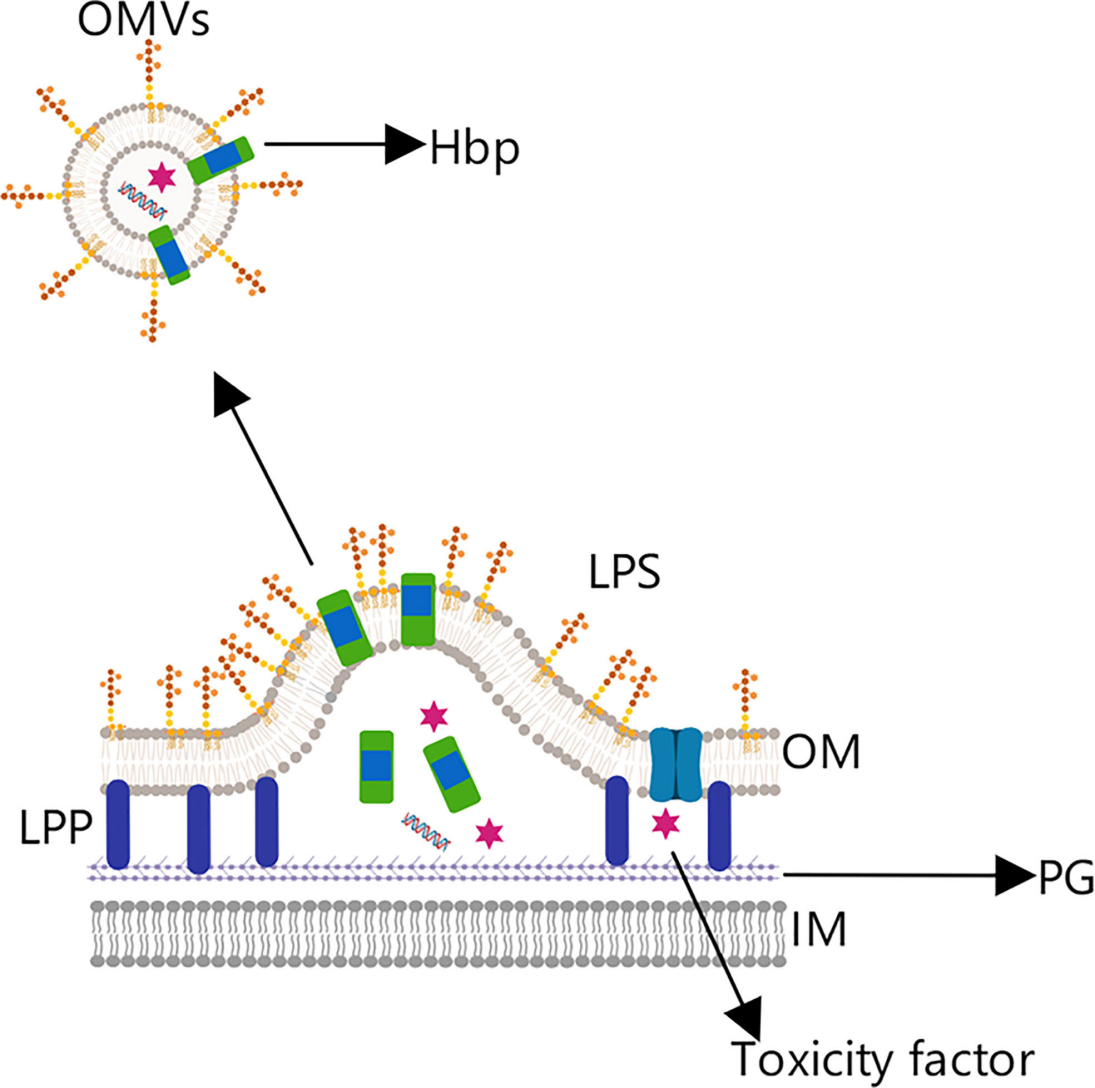
OMV tumor vaccine based on the hemoglobin protein. Hemoglobin protein (Hbp) is a virulence factor in Gram-negative bacteria that is transported to the outer membrane by the self-transport pathway.

Besides ClyA, the hemoglobin (Hbp) protein from *Escherichia coli* and meningococcal bacteria has also been used in the development of tumor vaccines (5, 71). Hbp is a virulence factor present in Gram-negative bacteria that can be secreted by a distinct transport pathway (**Figure 8**). Its protein structure includes a C-terminal helical domain, which helps locate the messenger domain in the outer membrane, an N-terminal signal peptide, which promotes protein transmembrane transport, and the messenger domain in the middle (71, 72).

Through genetic engineering technology, the native messenger domain can be replaced by a gene encoding a cancer antigen, allowing the antigen to be expressed on the OMV surface (72). For example, Matthias et al. effectively expressed *Mycobacterium tuberculosis* antigens (EASAT6, Rv2660c, and Ag85B) in OMVs produced by attenuated *Salmonella typhimurium*, and further demonstrated the Ag85B antigen so presented to be recognized and processed by DCs, ultimately inducing a specific cellular immune response (73).

However, at present, only OMV vaccines against meningococcal Group B have been successfully developed using this method, though they have demonstrated the ability to safely and effectively induce high levels of immune response in adult subjects and excellent immune protection in infants (74, 75). Most relevant OMV-based vaccines are currently in clinical trials, and extensive trials and data studies still remain to be carried out before they can be approved for marketing; thus, there are yet many wrinkles to be overcome concerning OMV-based anti-tumor vaccines.

### Limitations of OMV-based tumor vaccines

Although OMVs have many advantages for the preparation of anti-tumor vaccines on account of their physiological characteristics, there are also some hurdles still to be overcome in the application of OMV-based vaccines to treatment.

(1) One is the toxicity of OMVs themselves. Because OMVs are secreted from bacteria, OMVs contain a variety of virulence factors that confer toxicity, especially the LPS component on the membrane. Some early studies treated OMVs with detergent to reduce their toxicity (73). However, such treatment has also been shown to remove some substances that contribute to OMV immunogenicity, such as lipoprotein, thereby reducing the adjuvant activity (76, 77).
Another approach that has been shown to reduce OMV toxicity is mutating the acyltransferase involved in lipid A biosynthesis so that LPS is five acetoxylation rather than six (74). In addition, Lee J.B. et al. showed that *Salmonella typhimurium* OMVs with low endotoxin could be obtained by knocking out the *msbB* gene (78).
(2) Another hindrance is OMV yield. Although the process of isolating and purifying OMVs is not complicated, yields of prepared OMVs are generally low. Studies have shown that in meningococcal bacteria, knockout of *rmpM*, which encodes a peptidoglycan related to the Lpp family, can increase the yield of OMVs; similarly, elimination of the *E. coli Lpp* gene can also increase yield (79). Studies in *Shigella* have also shown that upon knockout of the *tolA* gene, not only is vesicle formation increased by about 60%, but also the immunogenicity is enhanced (80).
(3) A third area with challenges to be addressed concerns the antigen presentation and immune response induced by OMVs. Different cancers call for different antigens, so it is necessary to learn how best to select and express them on the OMV surface; moreover, OMVs secreted by some bacteria can have an immunosuppressive effect and interfere with the functions of protective immune molecules (54). How to reduce and eliminate such negative effects without also affecting the size and immunogenicity of OMVs is a key consideration in improving clinical treatment efficiency and reducing the risk posed by the vaccine itself.

In addition, the expression level of antigens is a concern; studies that prepared OMV-based vaccines for breast cancer have shown that the relevant antigen is expressed on only 1% of the OMV surface (5). Although this level of expression is sufficient to activate the immune response, increasing expression can help transmit more antigen to APCs and thereby enhance the response. Further, the composition of OMVs may result in the host body producing an unexpected response, which can overshadow the desired immune response and reduce the specific response to the target antigen (81). Therefore, it is necessary to strike a balance between eliciting the immune response and the efficiency of antigen presentation (5).

## Discussion

Group B meningococcal-associated OMV vaccines have achieved great success, and their safety and treatment efficiency have also been fully confirmed; consequently, OMV vaccines are receiving more and more research attention. The development of anti-tumor vaccines based on OMVs mainly exploits the capability of OMVs to carry proteins and small nucleic acids, their high immunogenicity, and their non-replication *in vitro*. With the help of gene recombination, gene knockout, and other technologies, antigens can be successfully introduced into OMVs and used to induce an immune response in the body. Continuing development of this technology can allow the formation of a commercial platform to further promote research into related applications. OMVs vaccine has better immunogenicity, does not need to protect against the degradation of the vaccine by host cells like RNA tumor vaccine and peptide vaccine, and avoids excessive consumption of vaccine and simplify the process of vaccine production. With the improvement of Plug-and-Display systems, OMVs themselves are able to present multiple antigens; vaccine epitope abundance increases; vaccine therapeutic efficacy increases, and there is no risk of new cancer introduction as whole-cell vaccines do. Therefore, OMVs vaccines have great advantages and prospects in terms of vaccine delivery, targeting, immunogenicity and antigen expression.

In addition, because OMVs are able to elicit congenital and adaptive immunity, they have the functionality of an adjuvant, and offer obvious advantages relative to aluminum adjuvants in the current market (82). Moreover, although studies have shown that membrane vesicles produced by Gram-positive bacteria such as *Clostridium* can trigger immune responses (83), the most studies to date have focused on OMVs generated by Gram-negative bacteria; thus, there remains considerable area for further study and improving our understanding of the physiological mechanisms involved.

To sum up, although we do not yet fully understand the physiological mechanisms of OMVs, this lack does not affect continuing exploration of their practical applications. As OMVs offer considerable advantages and good prospects in the field of anti-tumor vaccines, we have reason to believe that with continuous study of the physiological characteristics of OMVs and the immune mechanisms they elicit, we can give full play to their potential applications and ultimately form a new vaccine carrier platform based on OMVs, which will make great contributions to the prevention and treatment of cancers.

## Author contributions

All authors carried out reference searching, read the manuscript and provided feedback. SW wrote the paper and provided the **Figures 1**–**8** and **Supplementary Tables 1**–**6**. HH provided valuable suggestions for the modification of pictures, paper and tables, and made modifications with JG and YB. All authors have read and approved the final submitted manuscript.

## Funding

This work was supported by the Natural Sciences Foundation of China (NO. 31860309, 32160836), Science and Technology Major Project of Inner Mongolia Autonomous Region of China (No. 2020ZD15), Inner Mongolia Key Laboratory for Molecular Regulation of the Cell (NO. 2021PT0002).

## Conflict of interest

The authors declare that the research was conducted in the absence of any commercial or financial relationships that could be construed as a potential conflict of interest.

## Publisher’s note

All claims expressed in this article are solely those of the authors and do not necessarily represent those of their affiliated organizations, or those of the publisher, the editors and the reviewers. Any product that may be evaluated in this article, or claim that may be made by its manufacturer, is not guaranteed or endorsed by the publisher.
