# Supplementary material for: Bacterial outer membrane vesicles as a candidate tumor vaccine platform
Source: Front Immunol. 2022 Sep 9;13:987419. doi: 10.3389/fimmu.2022.987419 (PMC9505906; doi:10.3389/fimmu.2022.987419)
Supplement: Supplementary file 1 [file Table_1.docx]

Supplementary Table 1：DNA tumor vaccine related clinical trials

|  | **NCT Number** | **Title** | **Status** | **Study Results** | **Conditions** | **Interventions** | **Characteristics** |
| --- | --- | --- | --- | --- | --- | --- | --- |
| 1 | NCT01493154 | Safety Study of HPV DNA Vaccine to Treat Head and Neck Cancer Patients | Terminated | No Results Available | • Head and Neck Cancer | • Biological: DNA Vaccine • Drug: Cyclophosphamide | Phase: Phase 1 |
| 2 | NCT00807781 | Mammaglobin-A DNA Vaccine for Metastatic Breast Cancer | Completed | No Results Available | • Metastatic Breast Cancer | • Biological: Mammaglobin-A DNA vaccine | Phase: Phase 1 |
| 3 | NCT03122106 | Neoantigen DNA Vaccine in Pancreatic Cancer Patients Following Surgical Resection and Adjuvant Chemotherapy | Active, not recruiting | No Results Available | • Pancreatic Cancer • Pancreas Cancer • Cancer of the Pancreas | • Biological: Personalized neo-antigen DNA vaccine  • Device: TDS-IM Electrode Array System  • Procedure: Peripheral blood draws | Phase: Phase 1 |
| 4 | NCT02348320 | Safety and Immunogenicity of a Personalized Polyepitope DNA Vaccine Strategy in Breast Cancer Patients With Persistent Triple- Negative Disease Following Neo-adjuvant Chemotherapy | Completed | No Results Available | • Triple Negative Breast Cancer • Triple-Negative Breast Cancer • Triple Negative Breast Neoplasms | • Biological: Personalized polyepitope DNA vaccine | Phase: Phase 1 |
| 5 | NCT04090528 | pTVG-HP DNA Vaccine With or Without pTVG- AR DNA Vaccine and Pembrolizumab in Patients With Castration-Resistant, Metastatic Pro-state Cancer | Recruiting | No Results Available | • Castration-resistant Prostate Cancer • Metastatic Cancer • Prostate Cancer | • Biological: pTVG-HP • Biological: pTVG-AR • Drug: Pembrolizumab | Phase: Phase 2 |
| 6 | NCT00859729 | Dose Finding Study of a DNA Vaccine Delivered With Intradermal Electroporation in Patients With Prostate Cancer | Completed | No Results Available | • Prostate Cancer | • Biological: pVAXrcPSAv53l (DNA encoding rhesus PSA) • Device: DERMA VAX™ intradermal DNA delivery system | Phase: • Phase 1 • Phase 2 |
| 7 | NCT01706458 | Provenge With or Without pTVG-HP DNA Booster Vaccine in Prostate Cancer | Completed | Has Results | • Prostate Cancer | • Biological: sipuleucel-T • Biological: DNA Vaccine | Phase: Phase 2 |
| 8 | NCT03532217 | Neoantigen DNA Vaccine in Combination With Nivolumab/Ipilimumab and PROSTVAC in Metastatic Hormone-Sensitive Prostate Cancer | Completed | No Results Available | • Metastatic Hormone-Sensitive Prostate Cancer | • Biological: PROSTVAC-V • Biological: PROSTVAC-F • Drug: Nivolumab • Drug: Ipilimumab • Biological: Neoantigen DNA vaccine • Device: TriGrid Delivery System • Procedure: Tumor biopsy • Procedure: Peripheral blood • Procedure: Fecal samples • Procedure: Leukapheresis | Phase: Phase 1 |
| 9 | NCT00849121 | Two-Arm Study of a DNA Vaccine Encoding Prostatic Acid Phosphatase (PAP) in Patients With Non-Metastatic Castrate-Resistant Prostate Cancer | Completed | Has Results | • Prostate Cancer | • Biological: pTVG-HP with rhGM-CSF | Phase: Phase 2 |
| 10 | NCT03199040 | Neoantigen DNA Vaccine Alone vs. Neoantigen DNA Vaccine Plus Durvalumab in Triple Negative Breast Cancer Patients Following Standard of Care Therapy | Active, not recruiting | No Results Available | • Triple Negative Breast Cancer • Triple Negative Breast Neoplasms • TNBC - Triple-Negative Breast Cancer • Triple-negative Breast Carcinoma | • Drug: Durvalumab • Biological: Neoantigen DNA vaccine • Device: TDS-IM system (Inchor Medical Systems) • Procedure: Peripheral blood draw | Phase: Phase 1 |
| 11 | NCT02204098 | Safety and Immune Response to a Mammaglobin-A DNA Vaccine In Breast Cancer Patients Undergoing Neoadjuvant Endocrine Therapy | Recruiting | No Results Available | • Breast Cancer • Breast Carcinoma • Malignant Neoplasm of Breast | • Biological: Mammaglobin-A DNA Vaccine • Procedure: Optional biopsy | Phase: Phase 1 |
| 12 | NCT03600350 | pTVG-HP and Nivolumab in Patients With Non- Metastatic PSA-Recurrent Prostate Cancer | Active, not recruiting | No Results Available | • Prostate Cancer | • Biological: pTVG-HP • Drug: Nivolumab • Drug: GM-CSF | Phase: Phase 2 |
| 13 | NCT02172911 | A Study of INO-3112 DNA Vaccine With Electroporation in Participants With Cervical Cancer | Completed | Has Results | • Cervical Cancer | • Biological: INO-3112 • Device: CELLECTRA™-5P | Phase: • Phase 1 • Phase 2 |
| 14 | NCT03988283 | Neoepitope-based Personalized DNA Vaccine Approach in Pediatric Patients With Recurrent Brain Tumors | Not yet recruiting | No Results Available | • Pediatric Recurrent Brain Tumor | • Biological: Personalized neoantigen DNA vaccine • Device: TDS-IM System • Procedure: Peripheral blood draw | Phase: Phase 1 |
| 15 | NCT00121173 | Vaccine Therapy in Preventing Cervical Cancer in Patients With Cervical Intraepithelial Neoplasia | Completed | Has Results | • Cervical Cancer • Precancerous Condition | • Biological: pNGVL4a-Sig/ E7(detox)/HSP70 DNA vaccine | Phase: • Phase 1 • Phase 2 |
| 16 | NCT01064375 | Safety Study of DNA Vaccine Delivered by Intradermal Electroporation to Treat Colorectal Cancer | Unknown status | No Results Available | • Colorectal Cancer | • Biological: tetwtCEA DNA (wt CEA with tetanus toxoid Th epitope) • Device: Derma Vax (electroporation device) • Biological: GM-CSF • Drug: Cyclophosphamide | Phase: • Phase 1 • Phase 2 |
| 17 | NCT02411786 | A Phase I Study of a DNA Vaccine Encoding Androgen Receptor Ligand-Binding Domain (AR LBD) +/-GMCSF | Completed | No Results Available | • Prostate Cancer | • Biological: pTVG-AR • Biological: gm-csf | Phase: Phase 1 |
| 18 | NCT04131413 | HPV DNA Vaccine Via Electroporation for HPV16 Positive Cervical Neoplasia | Recruiting | No Results Available | • Human Papillomavirus Type 16 • Cervical Intraepithelial Neoplasia Grade II • Cervical Intraepithelial Neoplasia, Grade III | • Drug: pNGVL4aCRTE6E7L2 | Phase: Phase 1 |
| 19 | NCT01486329 | VXM01 Phase I Dose Escalation Study in Patients With Locally Advanced, Inoperable and Stage IV Pancreatic Cancer | Completed | No Results Available | • Stage IV Pancreatic Cancer | • Biological: VXM01 • Biological: Placebo | Phase: Phase 1 |
| 20 | NCT01304524 | A Study of VGX-3100 DNA Vaccine With Electroporation in Patients With Cervical Intraepithelial Neoplasia Grade 2/3 or 3 | Completed | No Results Available | • Cervical Intraepithelial Neoplasia | • Biological: VGX 3100 • Biological: Placebo • Device: CELLECTRA™-5P | Phase: Phase 2 |
| 21 | NCT04397003 | Personalized Neoantigen Vaccine in Combination With Durvalumab (MEDI4736) in Extensive Stage Small Cell Lung Cancer | Recruiting | No Results Available | • Extensive-stage Small Cell Lung Cancer | • Biological: Neoantigen DNA vaccine • Drug: Durvalumab • Device: TDS-IM v2.0 Device • Procedure: Peripheral blood draws | Phase: Phase 2 |
| 22 | NCT02499835 | Vaccine Therapy and Pembrolizumab in Treating Patients With Hormone-Resistant, Metastatic Prostate Cancer | Active, not recruiting | No Results Available | • Hormone-Resistant Prostate Cancer • Metastatic Malignant Neoplasm in the Bone • Metastatic Malignant Neoplasm in the Soft Tissues • Metastatic Prostate Carcinoma • Prostate Adenocarcinoma • Recurrent Prostate Carcinoma • Stage IV Prostate Cancer | • Biological: Pembrolizumab • Biological: pTVG-HP Plasmid DNA Vaccine | Phase: • Phase 1 • Phase 2 |
| 23 | NCT01322802 | Vaccine Therapy in Treating Patients With Stage III-IV or Recurrent Ovarian Cancer | Completed | No Results Available | • Stage III Ovarian Epithelial Cancer • Stage III Ovarian Germ Cell Tumor • Stage IV Ovarian Epithelial Cancer • Stage IV Ovarian Germ Cell Tumor | • Biological: pUMVC3-hIGFBP-2 multi-epitope plasmid DNA vaccine • Other: laboratory biomarker analysis | Phase: Phase 1 |
| 24 | NCT05242965 | A Multiple Antigen Vaccine (STEMVAC) for the Treatment of Patients With Stage IV Non- Squamous Non-Small Cell Lung Cancer | Not yet recruiting | No Results Available | • Lung Non-Squamous Non- Small Cell Carcinoma  • Stage IV Lung Cancer AJCC v8  • Stage IVA Lung Cancer AJCC v8  • Stage IVB Lung Cancer AJCC v8 | • Biological: CD105/Yb-1/SOX2/ CDH3/MDM2-polyepitope Plasmid DNA Vaccine • Biological: Sargramostim | Phase: Phase 2 |
| 25 | NCT01341652 | Phase II PAP Plus GM-CSF Versus GM-CSF Alone for Non-metastatic Prostate Cancer | Completed | Has Results | • Prostate Cancer | • Biological: pTVG-HP • Biological: rhGM-CSF | Phase: Phase 2 |
| 26 | NCT02529930 | An Exploratory Safety and Immunogenicity Study of HPV16+ Immunotherapy VB10.16 in Women With HSIL; CIN 2/3) | Completed | No Results Available | • High Grade Cervical Intraepithelial Neoplasia | • Biological: VB10.16 Immunotherapy (DNA vaccine) | Phase: • Phase 1 • Phase 2 |
| 27 | NCT04989946 | Androgen Deprivation, With or Without pTVG- AR, and With or Without Nivolumab, in Patients With Newly Diagnosed, High-Risk Prostate Cancer | Recruiting | No Results Available | • Prostate Cancer | • Drug: Degarelix • Biological: pTVG-AR • Drug: Nivolumab | Phase: • Phase 1 • Phase 2 |
| 28 | NCT00096629 | Vaccine Therapy in Treating Patients With Kidney Cancer | Completed | No Results Available | • Kidney Cancer | • Biological: human prostate- specific membrane antigen plasmid DNA vaccine • Biological: mouse prostate- specific membrane antigen plasmid DNA vaccine | Phase: Phase 1 |
| 29 | NCT01334060 | WT1 Immunity Via DNA Fusion Gene Vaccination in Haematological Malignancies by Intramuscular Injection Followed by Intramuscular Electroporation | Completed | No Results Available | • Leukaemia (Acute) • Leukaemia (Chronic) • Leukaemia (Acute Myeloid) • Leukaemia (Acute Lymphoblastic) • Leukaemia (Acute Promyelocytic) | • Biological: p.DOM-WT1-37 DNA Vaccine and p.DOM- WT1-126 DNA Vaccine | Phase: Phase 2 |
| 30 | NCT03914872 | Expanded Access Neoantigen Vaccine in Solid Tumors | Temporarily not available | No Results Available | • Solid Tumor, Adult | • Biological: Neoantigen DNA Vaccine • Device: Integrated electroporation device |  |
| 31 | NCT02157051 | Vaccine Therapy in Treating Patients With HER2-Negative Stage III-IV Breast Cancer | Active, not recruiting | No Results Available | • HER2 Negative Breast Carcinoma • Recurrent Breast Carcinoma • Stage IIIA Breast Cancer • Stage IIIB Breast Cancer • Stage IIIC Breast Cancer • Stage IV Breast Cancer • Stage III Breast Cancer | • Biological: CD105/Yb-1/SOX2/ CDH3/MDM2-polyepitope Plasmid DNA Vaccine • Other: Laboratory Biomarker Analysis | Phase: Phase 1 |
| 32 | NCT00988559 | Therapeutic Vaccination for Patients With HPV16+ Cervical Intraepithelial Neoplasia (CIN2/3) | Completed | Has Results | • HPV16 Positive • Cervical Intraepithelial Neoplasia (CIN 2/3) | • Biological: DNA vaccination • Device: Gene gun vaccine • Biological: intramuscular vaccination • Biological: intra-lesional vaccine administration • Procedure: therapeutic resection of the lesion • Drug: imiquimod | Phase: Phase 1 |
| 33 | NCT05455658 | STEMVAC in Patients With Early Stage Triple Negative Breast Cancer | Not yet recruiting | No Results Available | • Anatomic Stage IB Breast Cancer AJCC v8 • Anatomic Stage II Breast Cancer AJCC v8 • Anatomic Stage IIA Breast Cancer AJCC v8 • Anatomic Stage IIB Breast Cancer AJCC v8 • Anatomic Stage III Breast Cancer AJCC v8 • Anatomic Stage IIIA Breast Cancer AJCC v8 • Anatomic Stage IIIB Breast Cancer AJCC v8 • Anatomic Stage IIIC Breast Cancer AJCC v8 • Prognostic Stage IB Breast Cancer AJCC v8 • Prognostic Stage II Breast Cancer AJCC v8 • and 7 more | • Biological: CD105/Yb-1/SOX2/ CDH3/MDM2-polyepitope Plasmid DNA Vaccine • Biological: Sargramostim | Phase: Phase 2 |
| 34 | NCT00582140 | Prostatic Acid Phosphatase (PAP) Vaccine in Patients With Prostate Cancer | Completed | No Results Available | • Prostate Cancer | • Biological: pTVG-HP with rhGM-CSF | Phase: Phase 1 |
| 35 | NCT00093548 | Vaccine Therapy in Treating Patients With Stage II, Stage IIIA, Stage IIIB, or Stage IVA Liver Cancer | Withdrawn | No Results Available | • Liver Cancer | • Biological: alpha fetoprotein adenoviral vector vaccine • Biological: alpha fetoprotein plasmid DNA vaccine • Biological: sargramostim plasmid DNA hepatocellular carcinoma vaccine adjuvant | Phase: • Phase 1 • Phase 2 |
