# Supplementary material for: Bacterial outer membrane vesicles as a candidate tumor vaccine platform
Source: Front Immunol. 2022 Sep 9;13:987419. doi: 10.3389/fimmu.2022.987419 (PMC9505906; doi:10.3389/fimmu.2022.987419)
Supplement: Supplementary file 2 [file Table_2.docx]

Supplementary Table 2：RNA tumor vaccine related clinical trials

|  | **NCT Number** | **Title** | **Status** | **Study Results** | **Conditions** | **Interventions** | **Characteristics** |
| --- | --- | --- | --- | --- | --- | --- | --- |
| 1 | NCT04932863 | BNT162b2 Messenger Ribonucleic Acid (mRNA) Covid-19 Vaccine in Cancer Patients on Active Treatment | Recruiting | No Results Available | • Neoplasms  • Cancer, Treatment-Related | • Biological: BNT162b2 mRNA Covid-19 Vaccine |  |
| 2 | NCT04163094 | Ovarian Cancer Treatment With a Liposome Formulated mRNA Vaccine in Combination With (Neo-)Adjuvant Chemotherapy | Active, not recruiting | No Results Available | • Ovarian Cancer | • Drug: W_ova1 Vaccine | Phase: Phase 1 |
| 3 | NCT03164772 | Phase 1/2 Study of Combination Immunotherapy and mRNA Vaccine in Subjects With NSCLC | Completed | No Results Available | • Metastatic Non-small Cell Lung Cancer • NSCLC | • Drug: Durvalumab • Drug: Tremelimumab • Biological: BI 1361849 | Phase: • Phase 1 • Phase 2 |
| 4 | NCT00831467 | Safety and Efficacy Trial of a RNActive®- Derived Prostate Cancer Vaccine in Hormone Refractory Disease | Completed | No Results Available | • Hormonal Refractory Prostate Cancer | • Biological: CV9103 | Phase: • Phase 1 • Phase 2 |
| 5 | NCT03908671 | Clinical Study of Personalized mRNA Vaccine Encoding Neoantigen in Patients With Advanced Esophageal Cancer and Non-small Cell Lung Cancer | Not yet recruiting | No Results Available | • Esophageal Cancer • Non Small Cell Lung Cancer | • Biological: Personalized mRNA Tumor Vaccine | Phase: Not Applicable |
| 6 | NCT03480152 | Messenger RNA (mRNA)-Based, Personalized Cancer Vaccine Against Neoantigens Expressed by the Autologous Cancer | Terminated | Has Results | • Melanoma • Colon Cancer • Gastrointestinal Cancer • Genitourinary Cancer • Hepatocellular Cancer | • Biological: National Cancer Institute (NCI)-4650, a messenger ribonucleic acid (mRNA)-based, Personalized Cancer Vaccine | Phase: • Phase 1 • Phase 2 |
| 7 | NCT05192460 | Safety and Efficacy of Personalized Neoantigen Vaccine in Advanced Gastric Cancer, Esophageal Cancer and Liver Cancer | Recruiting | No Results Available | • Gastric Cancer • Esophageal Cancer • Liver Cancer | • Biological: neoantigen tumor vaccine with or without PD-1/L1 | Phase: Not Applicable |
| 8 | NCT02316457 | RNA-Immunotherapy of IVAC_W_bre1_uID and IVAC_M_uID | Active, not recruiting | No Results Available | • Breast Cancer (Triple Negative Breast Cancer (TNBC)) | • Biological: IVAC_W_bre1_uID • Biological: IVAC_W_bre1_uID/ IVAC_M_uID | Phase: Phase 1 |
| 9 | NCT05359354 | Safety and Efficacy of Personalized Neoantigen Vaccine in Advanced Solid Tumors | Not yet recruiting | No Results Available | • Solid Tumor | • Biological: Personalized neoantigen tumor vaccine | Phase: Not Applicable |
| 10 | NCT04878796 | Effectiveness of mRNA Covid-19 Vaccines on Cancer Patients:Observational Study. (ANTICOV) | Recruiting | No Results Available | • Cancer • Covid-19 • Vaccine Response | • Diagnostic Test: Serum collection, adverse events collection |  |
| 11 | NCT03418480 | HPV Anti-CD40 RNA Vaccine | Recruiting | No Results Available | • Carcinoma, Squamous Cell  • Head and Neck Neoplasm  • Cervical Neoplasm  • Penile Neoplasms Malignant | • Drug: BNT113 | Phase: • Phase 1 • Phase 2 |
| 12 | NCT05202561 | A Study of RNA Tumor Vaccine in Patients With Advanced Solid Tumors | Recruiting | No Results Available | • Advanced Solid Tumor | • Biological: RNA tumor vaccine • Biological: RNA tumor vaccine +Navuliumab | Phase: Phase 1 |
| 13 | NCT04951323 | Impact of the Immune System on Response to Anti-Coronavirus Disease 19 (COVID-19) Vaccine in Allogeneic Stem Cell Recipients (Covid Vaccin Allo) | Recruiting | No Results Available | • Coronavirus Disease 2019 (Covid19)  • Hematopoietic Neoplasms | • Drug: anti-COVID19 mRNA- based vaccine (BNT162b2, Comirnaty®, commercialized by Pfizer) | Phase: Phase 3 |
| 14 | NCT05119738 | Immune Response to Third Dose of SARS- CoV-2 Vaccine in a Cohort of Cancer Patients on Active Treatment | Recruiting | No Results Available | • Sars-CoV-2 Infection | • Biological: Three doses of BNT162b2 (observational)  • Biological: Two doses of Coronavac and one dose BNT162b2 (observational) |  |
| 15 | NCT04935528 | Mechanisms of Anti COVID-19 Humoral and Cellular Immune Response After Vaccination in a Sample of Patients and Salaried Staff From a French Anti-cancer Center | Recruiting | No Results Available | • Cancer | • Biological: serology and ELISPOT test • Biological: serology | Phase: Not Applicable |
| 16 | NCT03468244 | Clinical Study of Personalized mRNA Vaccine Encoding Neoantigen in Patients With Advanced Digestive System Neoplasms | Unknown status | No Results Available | • Advanced Esophageal Squamous Carcinoma • Gastric Adenocarcinoma • Pancreatic Adenocarcinoma • Colorectal Adenocarcinoma | • Biological: Personalized mRNA Tumor Vaccine | Phase: Not Applicable |
| 17 | NCT05016622 | Booster Dose Trial | Recruiting | No Results Available | • Cancer | • Biological: BNT162b2 vaccine | Phase: Phase 2 |
| 18 | NCT05270967 | FDG (Fluorodeoxyglucose) Findings After COVID-19 Vaccination | Completed | No Results Available | • Vaccine Reaction • Oncology | • Diagnostic Test: F-18 FDG PET/CT imaging • Biological: Pfizer/biontech mRNA vaccine |  |
| 19 | NCT05028374 | COVID-19 VAX Booster Dosing in Patients With Hematologic Malignancies | Recruiting | No Results Available | • Multiple Myeloma • AL Amyloidosis • Chronic Lymphocytic Leukemia | • Drug: A single "booster" dose of the Moderna mRNA COVID-19 vaccine | Phase: Phase 2 |
| 20 | NCT02410733 | Evaluation of the Safety and Tolerability of i.v. Administration of a Cancer Vaccine in Patients With Advanced Melanoma | Active, not recruiting | No Results Available | • Melanoma | • Biological: Lipo-MERIT | Phase: Phase 1 |
| 21 | NCT04534205 | A Clinical Trial Investigating the Safety, Tolerability, and Therapeutic Effects of BNT113 in Combination With Pembrolizumab Versus Pembrolizumab Alone for Patients With a Form of Head and Neck Cancer Positive for Human Papilloma Virus 16 and Expressing the Protein PD-L1 | Recruiting | No Results Available | • Unresectable Head and Neck Squamous Cell Carcinoma • Metastatic Head and Neck Cancer • Recurrent Head and Neck Cancer | • Biological: BNT113 • Biological: Pembrolizumab | Phase: Phase 2 |
| 22 | NCT00204516 | Vaccination With Tumor mRNA in Metastatic Melanoma - Fixed Combination Versus Individual Selection of Targeted Antigens | Completed | No Results Available | • Malignant Melanoma | • Biological: mRNA coding for melanoma associated antigens • Drug: GM-CSF | Phase: • Phase 1 • Phase 2 |
| 23 | NCT00108264 | Tumor RNA Transfected Dendritic Cell Vaccines | Completed | No Results Available | • Prostate Cancer | • Biological: Tumor RNA transfected dendritic cells | Phase: Phase 1 |
| 24 | NCT00003432 | Immunotherapy in Treating Patients With Metastatic Breast Cancer | Terminated | No Results Available | • Breast Cancer | • Biological: carcinoembryonic antigen RNA-pulsed DC cancer vaccine | Phase: • Phase 1 • Phase 2 |
| 25 | NCT00004211 | Vaccine Therapy in Treating Patients With Metastatic Prostate Cancer | Completed | No Results Available | • Prostate Cancer | • Biological: PSA RNA-pulsed dendritic cell vaccine | Phase: • Phase 1 • Phase 2 |
| 26 | NCT01153113 | Human Telomerase Reverse Transcriptase Messenger RNA (hTERT mRNA) Transfected Dendritic Cell Vaccines | Withdrawn | No Results Available | • Metastatic Prostate Cancer | • Biological: hTERT mRNA DC | Phase: • Phase 1 • Phase 2 |
| 27 | NCT04784689 | Host Immune Response to Novel RNA COVID-19 Vaccination | Recruiting | No Results Available | • COVID-19 Vaccine • Cancer | • Biological: COVID-19 Vaccine |  |
| 28 | NCT04862806 | Safety, Efficacy of BNT162b2 mRNA Vaccine in CLL | Recruiting | No Results Available | • Chronic Lymphocytic Leukemia | • Diagnostic Test: COVID-19 serology | Phase: Not Applicable |
| 29 | NCT00004604 | Biological Therapy in Treating Patients With Metastatic Cancer | Completed | No Results Available | • Breast Cancer • Colorectal Cancer • Extrahepatic Bile Duct Cancer • Gallbladder Cancer • Gastric Cancer • Head and Neck Cancer • Liver Cancer • Lung Cancer • Metastatic Cancer • Ovarian Cancer • Pancreatic Cancer • Testicular Germ Cell Tumor | • Biological: CEA RNA-pulsed DC cancer vaccine | Phase: Phase 1 |
| 30 | NCT00087984 | RNA-Loaded Dendritic Cell Cancer Vaccine | Completed | No Results Available | • Renal Cell Carcinoma | • Biological: MB-002 | Phase: • Phase 1 • Phase 2 |
| 31 | NCT00005816 | Vaccine Therapy in Treating Patients With Stage III or Stage IV Kidney Cancer | Completed | No Results Available | • Kidney Cancer | • Biological: therapeutic autologous dendritic cells • Procedure: conventional surgery | Phase: Phase 1 |
| 32 | NCT00010127 | Vaccine Therapy in Treating Patients With Metastatic Prostate Cancer | Terminated | No Results Available | • Prostate Cancer | • Biological: therapeutic autologous dendritic cells | Phase: Phase 1 |
| 33 | NCT04847050 | A Trial of the Safety and Immunogenicity of the COVID-19 Vaccine (mRNA-1273) in Participants With Hematologic Malignancies and Various Regimens of Immunosuppression, and in Participants With Solid Tumors on PD1/ PDL1 Inhibitor Therapy, Including Boost... | Recruiting | No Results Available | • Solid Tumor Malignancy • Hematologic Malignancy • Leukemia • Lymphoma • Multiple Myeloma | • Biological: mRNA-1273 | Phase: Phase 2 |
| 34 | NCT02170389 | Vaccine Therapy Before Surgery in Treating Patients With Localized Kidney Cancer | Terminated | Has Results | • Recurrent Renal Cell Carcinoma • Stage I Renal Cell Cancer • Stage II Renal Cell Cancer | • Other: Laboratory Biomarker Analysis • Procedure: Nephrectomy • Biological: Renal Cell Carcinoma/CD40L RNA- Transfected Autologous Dendritic Cell Vaccine AGS-003 | Phase: Not Applicable |
| 35 | NCT05456165 | Study of an Individualized Vaccine Targeting Neoantigens in Combination With Immune Checkpoint Blockade for Patients With Colon Cancer | Recruiting | No Results Available | • Colonic Neoplasms • Colorectal Neoplasms | • Drug: GRT-C901 • Drug: GRT-R902 • Drug: Atezolizumab • Drug: Ipilimumab • Drug: Adjuvant chemotherapy | Phase: Phase 2 |
| 36 | NCT04487756 | Combination of Atezolizumab With Dendritic Cell Vaccine in Patients With Lung Cancer | Recruiting | No Results Available | • Extensive-stage Small Cell Lung Cancer | • Drug: Atezolizumab 1200 mg in 20 ML Injection • Biological: ADC Vaccine • Drug: Carboplatin | Phase: • Phase 1 • Phase 2 |
| 37 | NCT00003433 | Immunotherapy in Treating Patients With Resected Liver Metastases From Colon Cancer | Completed | No Results Available | • Colorectal Cancer • Metastatic Cancer | • Biological: carcinoembryonic antigen RNA-pulsed DC cancer vaccine | Phase: • Phase 1 • Phase 2 |
| 38 | NCT00890032 | Vaccine Therapy in Treating Patients Undergoing Surgery for Recurrent Glioblastoma Multiforme | Completed | No Results Available | • Recurrent Central Nervous System Neoplasm | • Biological: BTSC mRNA-loaded DCs | Phase: Phase 1 |
| 39 | NCT04998474 | FRAME-001 Personalized Vaccine in NSCLC | Not yet recruiting | No Results Available | • Non Small Cell Lung Cancer | • Biological: FRAME-001 personalized vaccine | Phase: Phase 2 |
| 40 | NCT00228189 | Carcinoembryonic Antigen-loaded Dendritic Cells in Advanced Colorectal Cancer Patients | Completed | No Results Available | • Colorectal Cancer • Liver Metastases | • Biological: CEA-loaded dendritic cell vaccine | Phase: • Phase 1 • Phase 2 |
| 41 | NCT01684241 | RBL001/RBL002 Phase I Clinical Trial | Completed | No Results Available | • Melanoma | • Biological: RBL001/RBL002 | Phase: Phase 1 |
