# Supplementary material for: Bacterial outer membrane vesicles as a candidate tumor vaccine platform
Source: Front Immunol. 2022 Sep 9;13:987419. doi: 10.3389/fimmu.2022.987419 (PMC9505906; doi:10.3389/fimmu.2022.987419)
Supplement: Supplementary file 3 [file Table_3.docx]

Supplementary Table 3：Peptide vaccine related clinical trials

|  | **NCT Number** | **Title** | **Status** | **Study Results** | **Conditions** | **Interventions** | **Characteristics** |
| --- | --- | --- | --- | --- | --- | --- | --- |
| 1 | NCT05013216 | Mutant KRAS -Targeted Long Peptide Vaccine for Patients at High Risk of Developing Pancreatic Cancer | Recruiting | No Results Available | • High Risk Cancer • Pancreatic Cancer | • Drug: KRAS peptide vaccine | Phase: Phase 1 |
| 2 | NCT04117087 | Pooled Mutant KRAS-Targeted Long Peptide Vaccine Combined With Nivolumab and Ipilimumab for Patients With Resected MMR-p Colorectal and Pancreatic Cancer | Recruiting | No Results Available | • Colorectal Cancer • Pancreatic Cancer | • Drug: KRAS peptide vaccine • Drug: Nivolumab • Drug: Ipilimumab | Phase: Phase 1 |
| 3 | NCT02019524 | Phase Ib Trial of Two Folate Binding Protein Peptide Vaccines (E39 and J65) in Breast and Ovarian Cancer Patients | Completed | No Results Available | • Breast Cancer • Ovarian Cancer | • Biological: E39 peptide vaccine • Biological: E39 vaccine then J65 vaccine • Biological: J65 vaccine then E39 vaccine | Phase: Phase 1 |
| 4 | NCT03956056 | Neoantigen Peptide Vaccine Strategy in Pancreatic Cancer Patients Following Surgical Resection and Adjuvant Chemotherapy | Active, not recruiting | No Results Available | • Pancreas Cancer • Pancreatic Cancer • Cancer of the Pancreas | • Biological: Neoantigen Peptide Vaccine • Drug: Poly ICLC • Procedure: Blood for immune monitoring | Phase: Phase 1 |
| 5 | NCT04024800 | Establishing the Recommended Biological Dose for AE37 Peptide Vaccine in Combination With Pembrolizumab That Will Enhance the Tumor-specific Immune Response and Demonstrate Efficacy in Patients With Advanced Triple-negative Breast Cancer | Active, not recruiting | No Results Available | • Triple-negative Breast Cancer | • Biological: AE37 Peptide vaccine • Biological: Pembrolizumab | Phase: Phase 2 |
| 6 | NCT00844506 | p53 Synthetic Long Peptides Vaccine With Cyclophosphamide for Ovarian Cancer | Completed | No Results Available | • Ovarian Cancer | • Drug: P53-SLP vaccine • Drug: Cyclophosphamide | Phase: Phase 2 |
| 7 | NCT01789099 | A Phase I/IIa Study of UV1 Vaccination in Patients With Non Small Cell Lung Cancer. | Active, not recruiting | No Results Available | • Non-small Cell Lung Cancer | • Biological: UV1 synthetic peptide vaccine and GM-CSF | Phase: • Phase 1 • Phase 2 |
| 8 | NCT01266720 | HLA-A*0201 Restricted Peptide Vaccine Therapy With Gemcitabine With Gemcitabine in Patient Pancreatic Cancer (Phase1) | Unknown status | No Results Available | • Pancreatic Cancer | • Biological: VEGFR1, VEGFR2 • Drug: Gemcitabine | Phase: Phase 1 |
| 9 | NCT04270149 | Cancer Peptides Plus GM-CSF and Adjuvant in Breast Cancer | Recruiting | No Results Available | • Breast Cancer | • Biological: ESR1 peptide vaccine | Phase: Phase 1 |
| 10 | NCT04397926 | Phase I Study of Individualized Neoantigen Peptides in the Treatment of EGFR Mutant Non-small Cell Lung Cancer | Recruiting | No Results Available | • Non Small Cell Lung Cancer | • Drug: Individualized neoantigen peptides vaccine | Phase: Phase 1 |
| 11 | NCT01784913 | A Phase I/IIa Study of UV1 Vaccine in Patients With Prostate Cancer | Active, not recruiting | No Results Available | • Prostate Cancer | • Biological: UV1 synthetic peptide vaccine and GM-CSF | Phase: • Phase 1 • Phase 2 |
| 12 | NCT00845611 | Human Leukocyte Antigen (HLA) - A*2402 Restricted Peptide Vaccine Therapy in Patients With Advanced Gastric Cancer | Completed | No Results Available | • Gastric Cancer | • Biological: peptide vaccine | Phase: Phase 1 |
| 13 | NCT05254184 | KRAS-Targeted Vaccine With Nivolumab and Ipilimumab for Patients With NSCLC | Not yet recruiting | No Results Available | • Non-Small Cell Lung Cancer | • Drug: Pooled Mutant KRAS- Targeted Long Peptide Vaccine 0.3mg each; 1.8mg total peptides | Phase: Phase 1 |
| 14 | NCT00561275 | Safety Study of Multiple Peptide Vaccine to Esophageal Cancer | Completed | No Results Available | • Esophageal Cancer | • Biological: LY6K, VEGFR1, VEGFR2 | Phase: Phase 1 |
| 15 | NCT00632333 | Combination of Chemoradiation Therapy and Epitope Peptide Vaccine Therapy in Treating Patients With Esophageal Cancer | Unknown status | No Results Available | • Esophageal Cancer | • Biological: URLC10, TTK, KOC1, VEGFR1, VEGFR2, cisplatin, fluorouracil | Phase: Phase 1 |
| 16 | NCT00892567 | Evaluating the T Cell Response to a Peptide- based Vaccine in Patients With Breast Cancer | Completed | No Results Available | • Breast Neoplasms | • Biological: 9 Peptides from Her-2/neu, CEA, & CTA | Phase: Phase 1 |
| 17 | NCT00437502 | A Phase I Study of Ovarian Cancer Peptides Plus GM-CSF and Adjuvant With Ovarian, Tubal or Peritoneal Cancer | Completed | No Results Available | • Epithelial Ovarian, Tubal or Peritoneal Cancer | • Biological: tumor peptide vaccine | Phase: Phase 1 |
| 18 | NCT04509167 | Pilot Study of Neoantigen Peptides for the Treatment of Neoplasms | Completed | No Results Available | • Neoplasms | • Biological: Neoantigen Peptides | Phase: Early Phase 1 |
| 19 | NCT05475106 | Pilot Study of Neoantigen Peptides and Leukine for the Treatment of Neoplasms | Recruiting | No Results Available | • Neoplasms | • Biological: Neoantigen Peptides | Phase: Early Phase 1 |
| 20 | NCT02427581 | Safety and Immunogenicity of a Personalized Synthetic Long Peptide Breast Cancer Vaccine Strategy in Patients With Persistent Triple- Negative Breast Cancer Following Neoadjuvant Chemotherapy | Withdrawn | No Results Available | • Triple Negative Breast Cancer • Triple Negative Breast Neoplasms • Triple-Negative Breast Cancer | • Biological: Personalized synthetic long peptide vaccine (Poly ICLC) • Drug: Poly ICLC | Phase: Phase 1 |
| 21 | NCT00616291 | Vaccine Therapy in Treating Patients With Metastatic, Progressive Prostate Cancer | Completed | No Results Available | • Prostate Cancer | • Biological: NY-ESO-1/LAGE-1 HLA class I/II peptide vaccine | Phase: Phase 1 |
| 22 | NCT00573495 | Multipeptide Vaccine for Advanced Breast Cancer | Completed | No Results Available | • Breast Neoplasm • Breast Cancer • Cancer of the Breast • Carcinoma, Ductal | • Biological: hTERT/Survivin Multi-Peptide Vaccine | Phase: Phase 1 |
| 23 | NCT03879694 | Survivin Long Peptide Vaccine in Treating Patients With Metastatic Neuroendocrine Tumors | Recruiting | No Results Available | • Lung Atypical Carcinoid Tumor • Lung Typical Carcinoid Tumor • Metastatic Pancreatic Neuroendocrine Tumor | • Biological: Incomplete Freund's Adjuvant • Drug: Octreotide Acetate • Biological: Sargramostim • Biological: SVN53-67/M57-KLH Peptide Vaccine | Phase: Phase 1 |
| 24 | NCT02593227 | Folate Receptor Alpha Peptide Vaccine With GM-CSF in Patients With Triple Negative Breast Cancer | Completed | No Results Available | • Breast Cancer | • Biological: Low dose FR# vaccine • Drug: Cyclophosphamide • Biological: High dose FR# vaccine | Phase: Phase 2 |
| 25 | NCT05010200 | The Safety and Tolerability of PGV001-based Personalized Multi-peptide Vaccines in the Adjuvant Setting. | Recruiting | No Results Available | • Prostate Cancer | • Biological: PGV-001 • Biological: Poly-ICLC • Biological: CDX-301 | Phase: Phase 1 |
| 26 | NCT00641615 | Peptide Vaccine and S-1/CPT-11 Therapy for Patients With Unresectable Advanced Colorectal Cancer | Completed | No Results Available | • Colorectal Cancer | • Biological: RNF43-721 | Phase: Phase 1 |
| 27 | NCT03784040 | Nivolumab, Ipilimumab and OTSGC-A24 Therapeutic Peptide Vaccine in Gastric Cancer - a Combination Immunotherapy Phase Ib Study. | Recruiting | No Results Available | • Gastric Cancer | • Drug: OTSGC-A24 • Drug: Nivolumab • Drug: Ipilimumab | Phase: Phase 1 |
| 28 | NCT05479045 | A Combination Therapy Strategy to Prevent Anti-PD-1 Therapy Resistance in Metastatic Ovarian Cancer Patients | Not yet recruiting | No Results Available | • Ovarian Cancer Stage IV • Ovarian Cancer Stage III • Ovarian Cancer Stage 3 | • Biological: NY-ESO-1 Peptide vaccine • Drug: Nivolumab | Phase: Phase 2 |
| 29 | NCT02128126 | Study of the Therapeutic Vaccine (ISA101/ ISA101b) to Treat Advanced or Recurrent Cervical Cancer | Completed | No Results Available | • Cervical Cancer | • Drug: ISA101/ISA101b | Phase: • Phase 1 • Phase 2 |
| 30 | NCT03068832 | Neoepitope-based Personalized Vaccine Approach in Pediatric Patients With Recurrent Brain Tumors | Withdrawn | No Results Available | • Pediatric Brain Tumor | • Biological: Personalized peptide vaccine • Drug: Poly ICLC • Procedure: Peripheral blood draw | Phase: Phase 1 |
| 31 | NCT00054912 | An Open Label Study of a Peptide Vaccine in Patients With Stage III Colon Cancer | Completed | No Results Available | • Colonic Neoplasms • Colorectal Neoplasms | • Biological: EP2101 | Phase: Phase 1 |
| 32 | NCT00874588 | Peptide Vaccine Targeting to Cancer Specific Antigen Combined With Anti-angiogenic Peptide Antigen in Treating Patients With Non- small Cell Lung Cancer | Completed | No Results Available | • Non Small Cell Lung Cancer | • Biological: HLA- A*2402restricted URLC10, CDCA1, VEGFR1 and VEGFR2 | Phase: Phase 1 |
