# Supplementary material for: Bacterial outer membrane vesicles as a candidate tumor vaccine platform
Source: Front Immunol. 2022 Sep 9;13:987419. doi: 10.3389/fimmu.2022.987419 (PMC9505906; doi:10.3389/fimmu.2022.987419)
Supplement: Supplementary file 4 [file Table_4.docx]

Supplementary Table 4：Tumor whole cell vaccine related clinical trials

|  | **NCT Number** | **Title** | **Status** | **Study Results** | **Conditions** | **Interventions** | **Characteristics** |
| --- | --- | --- | --- | --- | --- | --- | --- |
| 1 | NCT02479230 | Type I-Polarized Autologous Dendritic Cell Vaccine With Tumor Blood Vessel Antigen- Derived Peptides in Metastatic Breast Cancer Patients | Completed | No Results Available | • Breast Cancer • Metastatic Breast Cancer | • Biological: tumor blood vessel antigen peptide-pulsed alpha- type-1 polarized dendritic cell vaccine • Drug: gemcitabine hydrochloride | Phase: Phase 1 |
| 2 | NCT00345293 | Dendritic Cell Vaccine Study (DC/PC3) for Prostate Cancer | Completed | Has Results | • Prostate Cancer | • Biological: autologous dendritic cell vaccine (DC/PC3) | Phase: • Phase 1 • Phase 2 |
| 3 | NCT00868114 | Direct Tumor Injection KLH-Pulsed Dendritic Cells in Unresectable Pancreatic Cancer | Terminated | No Results Available | • Metastatic Pancreatic Cancer | • Biological: KLH-pulsed autologous dendritic cell vaccine | Phase: Phase 2 |
| 4 | NCT02115958 | The Immunotherapy of Nasopharyngeal Cancer Using Cancer Stem Cells Vaccine | Completed | No Results Available | • Neoplasms, Lung | • Biological: cancer stem cell vaccine | Phase: • Phase 1 • Phase 2 |
| 5 | NCT02074046 | Safety Study of Cancer Stem Cell Vaccine to Treat Pancreatic Cancer | Completed | No Results Available | • Neoplasms, Pancreas | • Biological: cancer stem cell vaccine | Phase: • Phase 1 • Phase 2 |
| 6 | NCT01398124 | Cyclin B1 Peptide-Pulsed Autologous Dendritic Cell Vaccine for Resectable Non-Small Cell Lung Cancer | Withdrawn | No Results Available | • Non-small Cell Lung Cancer | • Biological: Cyclin B1 Peptide | Phase: Not Applicable |
| 7 | NCT02176746 | A Phase I/II Study of Active Immunotherapy With Cancer Stem Cells Vaccine for Colorectal Cancer | Completed | No Results Available | • Neoplasms,Colorectal | • Biological: cancer stem cell vaccine | Phase: • Phase 1 • Phase 2 |
| 8 | NCT02084823 | Vaccine Therapy in Treating Lung Cancer Patients With Cancer Stem Cells | Completed | No Results Available | • Neoplasms, Lung | • Biological: cancer stem cell vaccine | Phase: • Phase 1 • Phase 2 |
| 9 | NCT02018458 | Safety Study Of Chemotherapy Combined With Dendritic Cell Vaccine to Treat Breast Cancer | Completed | Has Results | • Breast Cancer | • Biological: LA TNBC: DC vaccine+Preop chemo • Biological: ER+/HER2-BC:DC vaccine+Preop chemo | Phase: • Phase 1 • Phase 2 |
| 10 | NCT00140387 | Prime-Boost Dose Scheduling Trial for Human GM-CSF Gene Transduced Irradiated Prostate Allogeneic Cancer Cell Vaccines (Allogeneic Prostate GVAX®) | Completed | No Results Available | • Prostate Cancer | • Biological: Immunotherapy allogeneic GM-CSF secreting cellular vaccine | Phase: • Phase 1 • Phase 2 |
| 11 | NCT02178670 | Safety and Effectivity Immunotherapy to Treat Ovarian Cancer With Cancer Stem Cells Vaccine | Completed | No Results Available | • Neoplasms,Ovarian | • Biological: CSC-DC | Phase: • Phase 1 • Phase 2 |
| 12 | NCT02063724 | HER-2 Pulsed DC Vaccine to Prevent Recurrence of Invasive Breast Cancer | Active, not recruiting | Has Results | • Breast Cancer | • Biological: HER-2 pulsed Dendritic Cell Vaccine | Phase: Phase 1 |
| 13 | NCT02548169 | Dendritic Cell Vaccine and Chemotherapy for Patients With Pancreatic Cancer | Terminated | No Results Available | • Pancreatic Cancer | • Biological: DC Vaccine + Standard of Care Chemotherapy | Phase: Phase 1 |
| 14 | NCT04348747 | Dendritic Cell Vaccines Against Her2/Her3 and Pembrolizumab for the Treatment of Brain Metastasis From Triple Negative Breast Cancer or HER2+ Breast Cancer | Recruiting | No Results Available | • Anatomic Stage IV Breast Cancer AJCC v8 • Metastatic Malignant Neoplasm in the Brain • Metastatic Triple-Negative Breast Carcinoma • Prognostic Stage IV Breast Cancer AJCC v8 | • Biological: Anti-HER2/HER3 Dendritic Cell Vaccine • Biological: Pembrolizumab | Phase: Phase 2 |
| 15 | NCT05378464 | Adoptive T Cell Therapy Following HER2- Pulsed Dendritic Cell Vaccine & Pepinemab / Trastuzumab in Patients w/ Metastatic HER2+ Breast Cancer | Recruiting | No Results Available | • HER2-positive Breast Cancer | • Biological: Dendritic Cell (DC1) Vaccine • Drug: Trastuzumab • Drug: Pepinemab • Biological: T-Cell therapy | Phase: Phase 1 |
| 16 | NCT00266110 | Vaccine Therapy, Trastuzumab, and Vinorelbine in Treating Patients With Locally Recurrent or Metastatic Breast Cancer | Completed | Has Results | • Breast Cancer | • Biological: sargramostim • Biological: therapeutic autologous dendritic cells • Biological: trastuzumab • Drug: vinorelbine ditartrate | Phase: Phase 2 |
| 17 | NCT00031564 | Phase II Study of a B7-1 Gene-Modified Autologous Tumor Cell Vaccine and Systemic IL-2 | Completed | No Results Available | • Kidney Cancer | • Biological: Interleukin-2 • Biological: B7-1 | Phase: Phase 2 |
| 18 | NCT04082182 | MIDRIX4-LUNG Dendritic Cell Vaccine in Patients With Metastatic Non-small Cell Lung Cancer | Active, not recruiting | No Results Available | • Non-small Cell Lung Cancer Metastatic | • Biological: Dendritic cell immunotherapy • Biological: Antigen-specific DTH • Biological: Control DTH | Phase: Phase 1 |
| 19 | NCT02089919 | Cancer Stem Cells Vaccine Therapy in Treating Hepatocellular Cancer Patients | Completed | No Results Available | • Neoplasms, Liver | • Biological: cancer stem cell vaccine | Phase: • Phase 1 • Phase 2 |
| 20 | NCT00148993 | Allogeneic Tumor Cell Vaccination in Patients With Solid Tumors | Withdrawn | No Results Available | • Metastatic Solid Tumors | • Biological: Tumor Cell Vaccine | Phase: • Phase 1 • Phase 2 |
| 21 | NCT01595321 | Pancreatic Tumor Cell Vaccine (GVAX), Low Dose Cyclophosphamide, Fractionated Stereotactic Body Radiation Therapy (SBRT), and FOLFIRINOX Chemotherapy in Patients With Resected Adenocarcinoma of the Pancreas | Active, not recruiting | No Results Available | • Pancreatic Cancer | • Drug: Cyclophosphamide • Biological: PANC 10.05 pcDNA-1/GM-Neo and PANC 6.03 pcDNA-1/GM-Neo vaccine • Radiation: Stereotactic Body Radiation (SBRT) • Drug: FOLFIRINOX | Phase: Not Applicable |
| 22 | NCT01088789 | A Trial of Boost Vaccinations of Pancreatic Tumor Cell Vaccine | Recruiting | No Results Available | • Pancreatic Cancer | • Biological: PANC 10.05 pcDNA-1/GM-Neo and PANC 6.03 pcDNA-1 neo vaccine. | Phase: Phase 2 |
| 23 | NCT01061840 | Trial of Bi-shRNA-furin and GMCSF Augmented Autologous Tumor Cell Vaccine for Advanced Cancer | Completed | No Results Available | • Ewings Sarcoma • Non Small Cell Lung Cancer • Liver Cancer | • Biological: Vigil™ | Phase: Phase 1 |
| 24 | NCT03300843 | Ability of a Dendritic Cell Vaccine to Immunize Melanoma or Epithelial Cancer Patients Against Defined Mutated Neoantigens Expressed by the Autologous Cancer | Terminated | Has Results | • Melanoma • Gastrointestinal Cancer • Breast Cancer • Ovarian Cancer • Pancreatic Cancer | • Biological: Peptide loaded dendritic cell vaccine | Phase: Phase 2 |
| 25 | NCT00140374 | Vaccination Priming and Vaccine Boosting Trial of Allogeneic Human GM-CSF Gene Transduced Irradiated Prostate Cancer Cell Vaccines (GVAX® Vaccine for Prostate Cancer) | Completed | No Results Available | • Prostate Cancer | • Biological: Immunotherapy allogeneic GM-CSF secreting cellular vaccine | Phase: • Phase 1 • Phase 2 |
| 26 | NCT02061423 | HER-2 Pulsed DC Vaccine to Prevent Recurrence of Invasive Breast Cancer Post Neoadjuvant Chemotherapy | Active, not recruiting | No Results Available | • Breast Cancer | • Biological: HER-2 pulsed Dendritic Cell Vaccine | Phase: Phase 1 |
| 27 | NCT00108264 | Tumor RNA Transfected Dendritic Cell Vaccines | Completed | No Results Available | • Prostate Cancer | • Biological: Tumor RNA transfected dendritic cells | Phase: Phase 1 |
| 28 | NCT00683241 | A Phase I Clinical Trial of Autologous Dendritic Cell Vaccine for Recurrent Ovarian or Primary Peritoneal Cancer | Completed | No Results Available | • Ovarian Cancer • Peritoneal Cancer | • Biological: DCVac-L | Phase: Phase 1 |
| 29 | NCT03387553 | HER2 Directed Dendritic Cell Vaccine During Neoadjuvant Therapy of HER2+Breast Cancer | Active, not recruiting | No Results Available | • Breast Cancer • Breast Cancer Female • Breast Cancer, Male • Invasive Breast Cancer • HER2-positive Breast Cancer • HER2 Positive Breast Carcinoma • Stage II Breast Cancer • Stage III Breast Cancer | • Biological: Dendritic Cell Vaccine (DC1) • Drug: Neoadjuvant Chemotherapy • Procedure: Curative Surgery | Phase: Early Phase 1 |
| 30 | NCT01966289 | SGI-110 in Combination With an Allogeneic Colon Cancer Cell Vaccine (GVAX) and Cyclophosphamide (CY) in Metastatic Colorectal Cancer (mCRC) | Completed | No Results Available | • Metastatic Colorectal Cancer | • Drug: CY • Biological: GVAX • Drug: SGI-110 | Phase: Phase 1 |
| 31 | NCT00656123 | Study of Colon GVAX and Cyclophosphamide in Patients With Metastatic Colorectal Cancer | Completed | No Results Available | • Colorectal Cancer • Metastatic Cancer | • Biological: Colon GVAX • Drug: cyclophosphamide | Phase: Phase 1 |
