# Supplementary material for: Bacterial outer membrane vesicles as a candidate tumor vaccine platform
Source: Front Immunol. 2022 Sep 9;13:987419. doi: 10.3389/fimmu.2022.987419 (PMC9505906; doi:10.3389/fimmu.2022.987419)
Supplement: Supplementary file 5 [file Table_5.docx]

Supplementary Table 5：Tumor virus vaccine related clinical trials

|  | **NCT Number** | **Title** | **Status** | **Study Results** | **Conditions** | **Interventions** | **Characteristic**  **s** |
| --- | --- | --- | --- | --- | --- | --- | --- |
| 1 | NCT00004029 | Vaccine Therapy in Treating Patients With Metastatic Prostate Cancer | Completed | No Results Available | • Prostate Cancer | • Biological: recombinant viral vaccine therapy • Biological: sargramostim | Phase: Phase 1 |
| 2 | NCT00227474 | S0348 Vaccine Therapy in Treating Patients With Stage IIIB or Stage IV Breast Cancer in Remission | Withdrawn | No Results Available | • Breast Cancer | • Drug: recombinant modified vaccinia Ankara-5T4 vaccine • Procedure: adjuvant therapy • Procedure: recombinant viral vaccine therapy | Phase: Phase 2 |
| 3 | NCT04379960 | Identification of Immunogenic Neo-epitopes for the Development of Personalised Pancreatic Cancer Vaccines | Unknown status | No Results Available | • The Initial Aim of This Project is to Perform in Vitro Validation of Neoepitope Candidates Selected From Available Mutanome Data | • Other: Peptides |  |
| 4 | NCT01094405 | Efficacy of Recombinant Epstein-Barr Virus (EBV) Vaccine in Patients With Nasopharyngeal Cancer Who Had Residual EBV DNA Load After Conventional Therapy | Completed | No Results Available | • Nasopharyngeal Cancer • Epstein-Barr Virus Infections | • Biological: Recombinant Epstein-Barr Virus (EBV) Vaccine | Phase: Phase 2 |
| 5 | NCT00828022 | Measles Vaccine in Patients With Measles Virus-Positive, Advanced Non-Small Cell Lung Cancer | Unknown status | No Results Available | • Non-Small Cell Lung Cancer • Measles | • Biological: attenuated measles vaccine | Phase: • Phase 1 • Phase 2 |
| 6 | NCT02275039 | p53MVA Vaccine and Gemcitabine Hydrochloride in Treating Patients With Recurrent Ovarian Epithelial Cancer | Completed | No Results Available | • Recurrent Ovarian Epithelial Cancer • Recurrent Fallopian Tube Carcinoma • Recurrent Primary Peritoneal Carcinoma | • Biological: modified vaccinia virus ankara vaccine expressing p53 • Drug: gemcitabine hydrochloride • Other: laboratory biomarker analysis | Phase: Phase 1 |
| 7 | NCT02888418 | Safety and Immunogenicity Study of Human Papilloma Virus Vaccine in Women Aged 9 to 30 and Men Aged 9 to 17 | Unknown status | No Results Available | • Cervical Cancer | • Biological: Tetravalent recombinant human papilloma virus vaccine (6,11,16,18 type) (Hansenula polymorpha) • Biological: placebo | Phase: Phase 1 |
| 8 | NCT01666782 | Study Comparing High-Dose Flu Vaccine to Standard Vaccine in Cancer Patients Less Than 65 Receiving Chemotherapy | Completed | Has Results | • Cancer • Influenza Viral Infections | • Biological: Standard Trivalent Influenza Vaccine • Biological: High-Dose Influenza Vaccine | Phase: Phase 2 |
| 9 | NCT01800071 | A Phase Ib Trial of MVA-EBNA1/LMP2 Vaccine in Nasopharyngeal Carcinoma | Completed | No Results Available | • Nasopharyngeal Cancer • Epstein Barr Virus Infections | • Drug: MVA-EBNA1/LMP2 vaccine | Phase: Phase 1 |
| 10 | NCT00964210 | Protecting Young Special Risk Females From Cervical Cancer Through Human Papilloma Virus (HPV) Vaccination | Completed | No Results Available | • Cervical Cancer | • Drug: Licensed quadrivalent HPV vaccine, Gardasil | Phase: Phase 3 |
| 11 | NCT01046227 | Antibody Titer Analysis After H1N1 Vaccination in Pediatric Haemato-oncology Patients | Unknown status | No Results Available | • Serology Analysis • Novel H1N1 Influenza Vaccination • Pediatric Haemato-oncology Patients | • Biological: Influenza A (H1N1) 2009 monovalent vaccine, inactivated |  |
| 12 | NCT03815942 | VAccination in Early and ADvanced Prostate caNCEr | Unknown status | No Results Available | • Intermediate Risk Prostate Cancer • Castration-resistant Prostate Cancer | • Biological: ChAdOx1-MVA 5T4 vaccine • Drug: Nivolumab Infusion [Opdivo] | Phase: • Phase 1 • Phase 2 |
| 13 | NCT01209338 | Acceptability and Feasibility of Human Papilloma Virus Vaccine | Unknown status | No Results Available | • Cervical Cancer | • Behavioral: Health education | Phase: Not Applicable |
| 14 | NCT04410874 | Imvamune Vaccine for the Treatment of Non- melanoma Skin Cancer | Recruiting | No Results Available | • Non-melanoma Skin Cancer • Basal Cell Carcinoma • Squamous Cell Carcinoma | • Biological: Imvamune | Phase: • Phase 1 • Phase 2 |
| 15 | NCT01356823 | Dose-Ranging Study of Recombinant Human Papillomavirus Virus 16/18 Bivalent Vaccine | Completed | No Results Available | • Cervical Intraepithelial Neoplasia • Cervical Cancer | • Biological: 30µg HPV • Biological: 60µg HPV • Biological: 90µg HPV • Biological: Hepatitis B vaccine | Phase: Phase 2 |
| 16 | NCT01031719 | Clinical Trial to Compare the Immunogenicity, Safety, and Tolerability of an Adjuvanted A(H1N1) Influenza Vaccine Versus Non- Adjuvanted A(H1N1) Influenza Vaccines in Patients With Invasive Solid Tumors | Completed | No Results Available | • H1N1 Influenza Virus • Invasive Solid Tumors | • Biological: adjuvanted A(H1N1) influenza vaccine • Biological: non-adjuvanted A(H1N1) influenza vaccine | Phase: Phase 3 |
| 17 | NCT00408590 | Recombinant Measles Virus Vaccine Therapy and Oncolytic Virus Therapy in Treating Patients With Progressive, Recurrent, or Refractory Ovarian Epithelial Cancer or Primary Peritoneal Cancer | Completed | Has Results | • Ovarian Cancer • Primary Peritoneal Cavity Cancer | • Biological: carcinoembryonic antigen-expressing measles virus • Biological: oncolytic measles virus encoding thyroidal sodium iodide symporter • Genetic: reverse transcriptase- polymerase chain reaction • Other: laboratory biomarker analysis | Phase: Phase 1 |
| 18 | NCT03141463 | Vvax001 Cancer Vaccine in (Pre) Malignant Cervical Lesions | Completed | No Results Available | • CIN 2/3 • Cervical Cancer | • Biological: Vvax001 therapeutic cancer vaccine | Phase: Phase 1 |
| 19 | NCT01147991 | Vaccine Therapy in Treating Patients With Epstein-Barr Virus-Related Cancer | Completed | No Results Available | • Gastric Cancer • Head and Neck Cancer • Lymphoma • Lymphoproliferative Disorder • Nonneoplastic Condition | • Biological: EBNA1 C-terminal/ LMP2 chimeric protein- expressing recombinant modified vaccinia Ankara vaccine • Other: laboratory biomarker analysis • Other: pharmacological study | Phase: Phase 1 |
| 20 | NCT04113902 | The Effects of Health Education About Human Papilloma Virus and Cervical Cancer Prevention on Knowledge, Attitudes, Beliefs and Behaviors | Completed | No Results Available | • Health Education | • Behavioral: Health Education | Phase: Not Applicable |
| 21 | NCT00128661 | Vaccine To Prevent Cervical Intraepithelial Neoplasia or Cervical Cancer in Younger Healthy Participants | Completed | Has Results | • Cervical Cancer • Precancerous Condition | • Biological: human papillomavirus 16/18 L1 virus- like particle/AS04 vaccine  • Biological: hepatitis A inactivated virus vaccine | Phase: Phase 3 |
| 22 | NCT01263327 | Clinical Trial of Recombinant Human Papillomavirus Virus 16/18 Bivalent Vaccine | Completed | No Results Available | • Cervical Cancer | • Biological: HPV 16/18 | Phase: Phase 1 |
| 23 | NCT03315975 | Flu Vaccine Responses in the Setting of Melanoma Treatment | Active, not recruiting | No Results Available | • Viral Vaccines | • Biological: Inactivated influenza vaccine | Phase: Phase 4 |
| 24 | NCT01460719 | A Study to Evaluate the Safety and Immunogenicity of Inactivated Varicella- Zoster Virus (VZV) Vaccine in Adults With Hematologic Malignancies (HM) Receiving Treatment With Anti-Cluster of Differentiation (CD) 20 Monoclonal Antibodies (V212-013) | Completed | Has Results | • Herpes Zoster | • Biological: V212 | Phase: Phase 1 |
| 25 | NCT01191684 | Vaccine Therapy in Treating Patients With Colorectal, Stomach, or Pancreatic Cancer | Completed | No Results Available | • Recurrent Colon Cancer • Recurrent Gastric Cancer • Recurrent Pancreatic Cancer • Recurrent Rectal Cancer • Stage III Colon Cancer • Stage III Gastric Cancer • Stage III Pancreatic Cancer • Stage III Rectal Cancer • Stage IV Colon Cancer • Stage IV Gastric Cancer • Stage IV Pancreatic Cancer • Stage IV Rectal Cancer | • Other: laboratory biomarker analysis • Other: enzyme-linked immunosorbent assay • Other: flow cytometry • Other: immunoenzyme technique • Biological: modified vaccinia virus ankara vaccine expressing p53 | Phase: Phase 1 |
| 26 | NCT00667563 | Vaccine Therapy in Preventing HPV in HIV- Positive Women in India | Completed | Has Results | • Cervical Cancer • Nonneoplastic Condition • Precancerous Condition | • Biological: quadrivalent human papillomavirus (types 6, 11, 16, 18) recombinant vaccine • Genetic: DNA analysis • Genetic: polymerase chain reaction • Other: cytology specimen collection procedure • Procedure: colposcopic biopsy | Phase: Phase 1 |
| 27 | NCT00003871 | PSA Vaccine Therapy in Treating Patients With Advanced Prostate Cancer | Completed | No Results Available | • Prostate Cancer | • Biological: fowlpox virus vaccine vector • Biological: recombinant vaccinia prostate-specific antigen vaccine | Phase: Phase 2 |
| 28 | NCT00112112 | Safety Study to Evaluate FluMist in Immunocompromised Children | Completed | Has Results | • Cancer | • Biological: FluMist • Biological: Placebo | Phase: Phase 1 |
| 29 | NCT05334706 | A Study to Assess the Reduction of Human Papillomavirus (HPV) Viral Infectivity and Transmission in HPV-Positive Women After Vaccination With 9vHPV (RIFT-HPV) | Not yet recruiting | No Results Available | • Cervical Intraepithelial Neoplasia Grade I/ II/ III (CIN I/ II/III) • Human Papillomavirus (HPV) Infections • High-risk HPV • HPV-16/ 18 | • Biological: Nonavalent HPV vaccine (9vHPV/Gardasil-9™). | Phase: Phase 2 |
| 30 | NCT02128126 | Study of the Therapeutic Vaccine (ISA101/ ISA101b) to Treat Advanced or Recurrent Cervical Cancer | Completed | No Results Available | • Cervical Cancer | • Drug: ISA101/ISA101b | Phase: • Phase 1 • Phase 2 |
| 31 | NCT00666107 | Pilot Study to Determine the Safety and Efficacy of Gardasil Against the Human Papilloma Virus (HPV) in HIV-infected Men | Unknown status | No Results Available | • Anal Cancer • HIV Infections | • Biological: Gardasil | Phase: Not Applicable |
| 32 | NCT01376505 | Vaccine Therapy in Treating Patients With Metastatic Solid Tumors | Recruiting | No Results Available | • Malignant Solid Tumour • Breast Cancer • Malignant Tumor of Colon • GIST • Ovarian Cancer | • Biological: HER-2 vaccine • Biological: Extension HER-2 vaccine trial at OBD | Phase: Phase 1 |
| 33 | NCT00316706 | Human Papilloma Virus (HPV) Vaccine Trial in Young Adolescent Women With GlaxoSmithKline Biologicals' (GSK Bio) HPV-16/18 Vaccine | Completed | Has Results | • Cervical Intraepithelial Neoplasia • Papillomavirus Infection | • Biological: GSK Biologicals' HPV-16/18 Vaccine (Cervarix™) • Biological: Havrix™ | Phase: Phase 3 |
| 34 | NCT00906750 | A Study of a Live Intranasal Influenza Vaccine in Children With Cancer | Completed | No Results Available | • Cancer | • Biological: FluMist • Biological: Inactivated influenza vaccine | Phase: Phase 1 |
