# Supplementary material for: Bacterial outer membrane vesicles as a candidate tumor vaccine platform
Source: Front Immunol. 2022 Sep 9;13:987419. doi: 10.3389/fimmu.2022.987419 (PMC9505906; doi:10.3389/fimmu.2022.987419)
Supplement: Supplementary file 6 [file Table_6.docx]

Supplementary Table 6：OMVs vaccine related clinical trials

|  | **NCT Number** | **Title** | **Status** | **Study Results** | **Conditions** | **Interventions** | **Characteristics** |
| --- | --- | --- | --- | --- | --- | --- | --- |
| 1 | NCT02946385 | Study to Assess the Immunological Long- term Persistence of Antibodies (Abs) 2 Years After GlaxoSmithKline (GSK) Meningococcal ABCWY Vaccination in the V102_15 (NCT02212457) and Response to a Booster in Adolescents | Completed | Has Results | • Infections, Meningococcal | • Biological: Meningococcal ABCWY Vaccine • Biological: Meningococcal B Recombinant vaccine | Phase: Phase 2 |
| 2 | NCT02451514 | A Study to Evaluate 4-year Antibody Persistence and Booster Response Following MenABCWY Vaccination in Healthy Adolescents and Young Adults Who Previously Participated in Studies V102_02 (NCT01210885) and V102_02E1 (NCT01367158) | Completed | Has Results | • Meningococcal Disease • Infections, Meningococcal | • Biological: MenABCWY+OMV vaccine | Phase: Phase 2 |
| 3 | NCT00774384 | Regulation of Mucosal Immune Response to Systemic MenB Vaccine | Completed | No Results Available | • Meningococcal Infections | • Biological: NZ MenB OMV vaccine (NZ98/254) | Phase: Phase 2 |
| 4 | NCT02212457 | Trial to Assess Immunogenicity and Safety of GlaxoSmithKline (GSK) Biologicals' Meningococcal ABCWY Vaccine as Compared to Meningococcal B Vaccine in Adolescents | Completed | Has Results | • Infections, Meningococcal | • Biological: Bexsero • Other: Saline Placebo • Biological: Havrix • Biological: MenABCWY | Phase: Phase 2 |
| 5 | NCT01621945 | Evaluation of Immune Response Against the Strain of Neisseria Meningitidis B: 14, P1-7, 16 in Patients Having to be Vaccinated MenBVac® (Extension EFFIVAC) | Completed | No Results Available | • Neisseria Meningitidis | • Biological: MenBvac |  |
| 6 | NCT01214850 | Novartis Vaccine and Diagnostics Carriage Trial | Completed | Has Results | • N. Meningitidis Carriage | • Biological: Meningococcal B Recombinant + Outer Membrane Vesicle vaccine (rMenB+OMV NZ) • Biological: MenACWY-CRM conjugate vaccine • Biological: Japanese Encephalitis vaccine | Phase: Phase 3 |
| 7 | NCT04502693 | Effectiveness of GlaxoSmithKline Biologicals S.A's Meningococcal Group B and Combined ABCWY Vaccines in Healthy Adolescents and Young Adults | Active, not recruiting | No Results Available | • Infections, Meningococcal | • Combination Product: rMenB +OMV NZ vaccine • Biological: Meningococcal Groups A, C, W and Y Conjugate Vaccine (MenACWY) • Combination Product: Placebo • Combination Product: MenABCWY-1 • Combination Product: MenABCWY-2 • Combination Product: MenABCWY-3 | Phase: Phase 3 |
| 8 | NCT01272180 | Safety and Immunogenicity of Different Meningitis Vaccine Formulations in Adolescents and Young Adults | Completed | Has Results | • Invasive Meningococcal Disease | • Biological: Meningococcal (groups A, C, W, Y) oligosaccharide diphtheria CRM-197 conjugate combined with meningococcal (group B) multicomponent recombinant vaccine + OMV. • Biological: Meningococcal (group B) multicomponent recombinant adsorbed vaccine plus OMV. • Biological: Meningococcal (groups A, C, W, and Y) oligosaccharide diphtheria CRM-197 conjugate vaccine. • Biological: Meningococcal (groups A, C, W, Y) oligosaccharide diphtheria CRM-197 conjugate combined with meningococcal (group B) multicomponent recombinant vaccine + qOMV. | Phase: Phase 2 |
| 9 | NCT01339923 | A Phase 3B, Open Label, Multi-Center Study to Evaluate the Safety, Tolerability and Immunogenicity of Novartis Meningococcal B Recombinant Vaccine When Administered Alone to Healthy Infants According to Different Immunization Schedules and to Healthy Children Aged 2 to 10 Years | Completed | Has Results | • Meningococcal Disease • Meningococcal Meningitis | • Biological: rMenB + OMV NZ vaccine • Biological: Meningococcal C oligosaccharide conjugated vaccine • Biological: Pneumococcal polysaccharide conjugate vaccine, 10 valent adsorbed. | Phase: Phase 3 |
| 10 | NCT00433914 | Safety, Tolerability and Immunogenicity of Two Different Formulations of Meningococcal B Recombinant Vaccine, When Administered to Healthy Infants | Completed | Has Results | • Meningococcal Disease | • Biological: rMenB • Biological: rMenB+OMV | Phase: Phase 2 |
| 11 | NCT01027351 | Extension Study Evaluating Antibody Persistence and Safety, Tolerability and Immunogenicity of Booster Doses of Novartis rMenB±OMV NZ Vaccine in Healthy UK Children Who Previously Received One or Four Doses of the Same Vaccine | Completed | Has Results | • Meningococcal Disease | • Biological: Meningococcal (group B) multicomponent recombinant adsorbed vaccine. • Biological: Meningococcal (group B) multicomponent recombinant adsorbed vaccine, without Outer Membrane Vesicles (OMV) | Phase: Phase 2 |
| 12 | NCT01478347 | A Phase 3b Study to Assess the Safety of Novartis Meningococcal B Recombinant Vaccine When Administered in Healthy At-risk Adults | Completed | Has Results | • Meningococcal Disease • Meningococcal Meningitis | • Biological: Recombinant meningococcal B + OMV NZ | Phase: Phase 3 |
| 13 | NCT01026974 | Extension Study Evaluating Antibody Persistence and Safety, Tolerability and Immunogenicity of a Booster Dose of Novartis rMenB±OMV NZ Vaccine in Healthy UK Children Who Previously Received Three Doses of the Same Vaccine | Completed | Has Results | • Meningococcal Disease | • Biological: rMenB • Biological: rMenB+OMV NZ | Phase: Phase 2 |
| 14 | NCT01423084 | Safety and Immunogenicity of Novartis Meningococcal B Vaccine Formulated With OMV Manufactured at Two Different Sites, in Healthy Adolescents Aged 11-17 Years | Completed | Has Results | • Meningococcal Disease • Meningococcal Meningitis | • Biological: Serogroup B meningococcal vaccine | Phase: Phase 3 |
| 15 | NCT01717638 | Persistence of Antibody Levels and Response to Fifth or Third Meningococcal B Recombinant Vaccine in 4-year Old Healthy Children Who Previously Participated in Study V72P12E1 | Completed | Has Results | • Meningococcal Disease • Meningococcal Meningitis | • Biological: 1 dose of Meningococcal (group B) multicomponent recombinant adsorbed vaccine • Biological: 2 doses of Meningococcal (group B) multicomponent recombinant adsorbed vaccine | Phase: Phase 3 |
| 16 | NCT01973218 | Safety and Immunogenicity Study of Two Doses of Novartis Meningococcal Serogroup B Recombinant Vaccine in Adolescents Aged 11-17 Years. | Completed | Has Results | • Meningococcal Disease | • Biological: Meningococcal B Recombinant vaccine rMenB +OMV NZ • Biological: Placebo • Biological: Meningococcal ACWY-CRM conjugate vaccine | Phase: Phase 3 |
| 17 | NCT00721396 | Safety, Tolerability and Immunogenicity of Meningococcal B Recombinant Vaccine Administered With or Without Routine Infant Vaccinations to Healthy Infants According to Different Immunization Schedules | Completed | Has Results | • Meningococcal Infections | • Biological: rMenB+OMV NZ • Biological: combined diphtheria,tetanus,pertussis +polio+Hepatitis B +Haemophilus influenzae B vaccine • Biological: Pneumococcal vaccine | Phase: Phase 2 |
| 18 | NCT01911221 | A Phase 3b, Single-Center, Open-label Study to Assess the Immunogenicity and Safety of Novartis Meningococcal B Recombinant Vaccine When Administered at a 0, 2-Month Schedule in Healthy At-Risk Adults Aged 18 to 65 Years Inclusive. | Completed | Has Results | • Prevention of the Meningococcal Disease | • Biological: rMenB+OMV NZ | Phase: Phase 3 |
| 19 | NCT04350138 | Safety and Efficacy Study of Meningococcal Group B Vaccine rMenB+OMV NZ (Bexsero) to Prevent Gonococcal Infection | Recruiting | No Results Available | • Gonococcal Infection | • Biological: Meningococcal Group B Vaccine • Other: Placebo | Phase: Phase 2 |
| 20 | NCT01148524 | Assessment of Antibody Persistence at Eighteen Months After the Completion of the Vaccination Course in Study V72P10 | Completed | Has Results | • Meningococcal Disease • Meningococcal Meningitis | • Biological: No Vaccine • Biological: rMenB+OMV-NZ | Phase: • Phase 2 • Phase 3 |
| 21 | NCT00381615 | Prevention, Randomized, Open Label, Active Control, Parallel Assignment, Safety/Efficacy Study | Completed | Has Results | • Healthy | • Biological: rMenB • Biological: rMenB+OMV • Biological: DTaP-Hib-IPV • Biological: PC7 • Biological: MenC-CRM • Biological: MenC-Hib • Biological: MMR | Phase: Phase 2 |
| 22 | NCT00944034 | Safety, Tolerability and Immunogenicity of Meningococcal B Recombinant Vaccine Administered as Booster Dose at 12, 18 or 24 Months of Age in Toddlers (12-24 Months) Primed With a Three-Dose Immunization Series as Infants in Study V72P12 | Completed | Has Results | • Meningococcal Disease • Meningococcal Meningitis | • Biological: rMenB+OMV NZ with routine vaccinations  • Biological: rMenB+OMV NZ  • Biological: two doses of rMenB  +OMV NZ | Phase: • Phase 2 • Phase 3 |
| 23 | NCT00661713 | Safety, Tolerability and Immunogenicity of Novartis Meningococcal B Recombinant Vaccine Administered to Healthy Adolescents According to Different Vaccination Schedules | Completed | Has Results | • Meningococcal Disease | • Biological: rMenB+OMV NZ • Biological: Placebo | Phase: • Phase 2 • Phase 3 |
| 24 | NCT02305446 | Safety and Blood Donations in Adults Vaccinated With rMenB+OMV NZ. | Completed | Has Results | • Meningitis, Meningococcal, Serogroup B | • Biological: Meningococcal (group B) multicomponent recombinant adsorbed vaccine | Phase: Phase 3 |
| 25 | NCT04318548 | Study to Assess the Immunogenicity and Safety of GSK's Meningococcal Group B Vaccine When Administered Concomitantly With GSK's Meningococcal MenACWY Conjugate Vaccine in Healthy Subjects of 16-18 Years of Age | Recruiting | No Results Available | • Infections, Meningococcal | • Combination Product: Meningococcal Group B Vaccine (GSK3536829A) (rMenB+OMV NZ) • Biological: Meningococcal MenACWY Conjugate Vaccine (GSK3536820A) (MenA lyo + MenCWY liquid) • Combination Product: Placebo | Phase:  Phase 3 |
| 26 | NCT01992536 | Phase 2, Observer-Blind, Placebo-Controlled, Randomized, Multi-Center Extension Study to Evaluate the Safety and Immunogenicity of a Booster Dose of a MenABCWY Vaccine  Administered 24 Months Following the Primary Series to Adolescents and Young Adults Who Participated in V102_03 (NCT01272180) | Completed | Has Results | • Meningococcal Disease | • Biological: MenABCWY+OMV • Biological: MenABCWY +¼OMV • Biological: Placebo | Phase: Phase 2 |
| 27 | NCT01139021 | One Year Antibody Persistence After a Fourth Dose Boost or Two Catch-Up Doses of Novartis Meningococcal B Recombinant  Vaccine Administered Starting From 12 Months of Age and Response to a Third Dose Boost or Two Catch-Up Doses Starting at 24 Months of Age | Completed | Has Results | • Meningococcal Disease | • Biological: rMenB+OMV NZ | Phase: Phase 3 |
| 28 | NCT03587207 | Study to Assess Potential Immune Interference When GlaxoSmithKline (GSK) Biologicals' MenABCWY Vaccine is Administered to Healthy Subjects Aged 10-25 Years | Completed | Has Results | • Meningitis, Meningococcal | • Biological: MenABCWY vaccine • Biological: rMenB+OMV NZ (Bexsero) vaccine • Biological: MenACWY (Menveo) vaccine | Phase: Phase 2 |
| 29 | NCT02106390 | Safety and Immunogenicity of GlaxoSmithKline Biologicals Meningococcal Group B Vaccine When Administered Concomitantly With GlaxoSmithKline Biologicals MenACWY Conjugate Vaccine to Healthy Infants | Completed | Has Results | • Infections, Meningococcal | • Biological: Meningococcal group B Vaccine, rMenB+OMV NZ • Biological: Meningococcal ACWY Conjugate Vaccine, MenACWY | Phase: Phase 3 |
| 30 | NCT00657709 | Immunogenicity, Safety and Lot to Lot Consistency of Novartis Meningococcal B Recombinant Vaccine When Administered With Routine Infant Vaccinations to Healthy Infants | Completed | Has Results | • Serogroup B Meningococcal Meningitis | • Biological: Serogroup B meningococcal Vaccine lot 1 (rMenB Lot 1) • Biological: Serogroup B meningococcal Vaccine lot 2 (rMenB Lot 2) • Biological: Serogroup B meningococcal Vaccine lot 3 (rMenB Lot 3)  • Biological: Infanrix Hexa  • Biological: Menjugate  • Biological: Prevenar | Phase: Phase 3 |
| 31 | NCT00297817 | Study of the Safety and Immune Response of Two Serogroup B Meningococcal Vaccines Administered to Healthy Adolescents | Completed | No Results Available | • Meningococcal Disease | • Biological: serogroup B meningococcal vaccine | Phase: Phase 2 |
| 32 | NCT00847145 | Extension Study of V72P13 to Evaluate the Safety, Tolerability and Immunogenicity of Novartis Meningococcal B Recombinant Vaccine When Administered as a Booster or as a Two-dose Catch-up to Healthy Toddlers | Completed | Has Results | • Meningococcal Disease | • Biological: 1a - rMenB+OMV NZ and routine vaccines • Biological: 1b - rMenB+OMV NZ and routine vaccines • Biological: 2a - Routine and rMenB+OMV NZ vaccines • Biological: 2b - rMenB+OMV NZ and routine vaccines • Biological: 3a - rMenB+OMV NZ and routine vaccines • Biological: 3b - 1 dose of rMenB+OMV NZ plus routine infant vaccinations • Biological: 4a- rMenB+OMV NZ and routine vaccines • Biological: 4b - rMenB+OMV NZ and routine vaccines | Phase: Phase 3 |
| 33 | NCT02141516 | Safety and Immunogenicity of Novartis Meningococcal B Vaccine When Administered to Immunocompromised Children and Adolescents Compared to Healthy Subjects. | Completed | Has Results | • Meningococcal Disease | • Biological: rMenB+OMV | Phase: Phase 3 |
| 34 | NCT02446743 | Combined Study - Phase 3b MenB Long Term Persistence in Adolescents | Completed | Has Results | • Infections, Meningococcal | • Biological: rMenB+OMV NZ (Meningococcal (Group B) multi component recombinant adsorbed vaccine) | Phase: Phase 3 |
| 35 | NCT03621670 | Safety and Immunogenicity of GSK Meningococcal Group B Vaccine and 13- valent Pneumococcal Vaccine Administered Concomitantly With Routine Infant Vaccines to Healthy Infants | Recruiting | No Results Available | • Infections, Meningococcal | • Biological: Bexsero (GSK Biologicals' Meningococcal group-B vaccine/ rMenB+OMV NZ) • Biological: Prevnar13 • Biological: Pediarix • Biological: Hiberix • Biological: Rotarix • Biological: M-M-R II • Biological: Varivax • Biological: Placebo (saline water) | Phase: Phase 3 |
| 36 | NCT04722003 | Mucosal Immunity Against Neisseria Gonorrhoeae After 4CMenB Vaccination | Recruiting | No Results Available | • Gonorrhoea | • Biological: Meningococcal Group B Vaccine • Other: Placebo | Phase: Phase 2 |
| 37 | NCT00443157 | Meningococcal B Vaccination in University Students | Completed | No Results Available | • Neisseria Meningitidis Serogroup B | • Biological: Meningococcal B Vaccine NZ | Phase: Phase 2 |
| 38 | NCT04297436 | Gonococcal Vaccine Study in Key Populations in Kenya | Completed | No Results Available | • Gonorrhea | • Biological: 4CMenB (Bexsero®) vaccine | Phase: Not Applicable |
| 39 | NCT02140762 | Effectiveness, Immunogenicity and Safety of Meningococcal ABCWY Vaccine Administered to Healthy Adolescents | Completed | Has Results | • Meningococcal Disease | • Biological: MenABCWY • Other: Placebo • Biological: MenACWY | Phase: Phase 2 |
| 40 | NCT02398396 | Investigating Meningococcal Vaccines in Adults | Completed | No Results Available | • Meningococcal Vaccines • Meningococcal Infections • Neisseria Meningitidis • Factor H-binding Protein, Neisseria Meningitidi | • Biological: 4CMenB (Bexsero®) - Novartis Vaccines and Diagnostics | Phase: Not Applicable |
| 41 | NCT01367158 | Safety, Tolerability and Immunogenicity of a Third Dose of One of Four Different Formulations of rMenB + MenACWY Combination Vaccine in Adolescents Who Previously Received the Same Study Vaccines | Completed | Has Results | • Meningococcal Disease • Meningococcal Meningitis | • Biological: Meningococcal (groups A, C, W, and Y) oligosaccharide diphtheria CRM-197 conjugate combined with meningococcal (group B) multicomponent recombinant vaccine • Biological: Meningococcal (group B) multicomponent recombinant adsorbed vaccine • Biological: Tdap | Phase: Phase 2 |
| 42 | NCT03493919 | A Sourcing Study to Collect Human Blood Samples From Healthy Adults | Completed | No Results Available | • Meningitis, Meningococcal | • Biological: Bexsero • Biological: Menveo | Phase: Phase 4 |
